# Characterization of the secretory profile and exosomes of limbal stem cells in the canine species

**DOI:** 10.1371/journal.pone.0244327

**Published:** 2020-12-29

**Authors:** Antonio J. Villatoro, Cristina Alcoholado, María del Carmen Martín-Astorga, Gustavo Rico, Viviana Fernández, José Becerra

**Affiliations:** 1 Laboratory of Bioengineering and Tissue Regeneration (LABRET), Department of Cell Biology, Genetics and Physiology, Faculty of Sciences, University of Málaga, IBIMA, Málaga, Spain; 2 Instituto de Immunología Clínica y Terapia Celular (IMMUNESTEM), Málaga, Spain; 3 Biomedicine Research Institute of Malaga (IBIMA), Campus Universitario Teatinos, Málaga, Spain; 4 Networking Research Center on Bioengineering, Biomaterials and Nanomedicine, (CIBER-BBN), Malaga, Spain; 5 Andalusian Centre for Nanomedicine and Biotechnology-BIONAND, Málaga, Spain; Cedars-Sinai Medical Center, UNITED STATES

## Abstract

Limbal stem cells (LSCs) are a quiescent cell population responsible for the renewal of the corneal epithelium. Their deficiency is responsible for the conjunctivization of the cornea that is seen in different ocular pathologies, both in humans and in the canine species. The canine species represents an interesting preclinical animal model in ocular surface pathologies. However, the role of LSCs in physiological and pathological conditions in canine species is not well understood. Our objective was to characterize for the first time the soluble factors and the proteomic profile of the secretome and exosomes of canine LSCs (cLSCs). In addition, given the important role that fibroblasts play in the repair of the ocular surface, we evaluated the influence of the secretome and exosomes of cLSCs on their proliferation in vitro. Our results demonstrated a secretory profile of cLSCs with high concentrations of MCP-1, IL-8, VEGF-A, and IL-10, as well as significant production of exosomes. Regarding the proteomic profile, 646 total proteins in the secretome and 356 in exosomes were involved in different biological processes. Functionally, the cLSC secretome showed an inhibitory effect on the proliferation of fibroblasts in vitro, which the exosomes did not. These results open the door to new studies on the possible use of the cLSC secretome or some of its components to treat certain pathologies of the ocular surface in canine species.

## Introduction

The cornea plays a fundamental role in clear vision. It has regeneration and repair capacity owing to the existence of a population of limbal stem cells (LSCs) residing within a cell niche in the transition zone between the cornea and the sclera (sclerocorneal limbus) in the so-called palisades of Vogt [1–3]. LSCs are a quiescent cell population with proliferative capacity that are responsible for the continuous renewal of the corneal epithelium, and their deficiency is responsible for corneal conjunctivization (vascularization, scarification, ulceration, chronic inflammation, and loss of corneal transparency), which triggers corneal opacity and blindness [4–6].

The LSC niche is one of the most active stem cell niches preserved in adulthood, characteristic of tissues that need continuous regeneration. This niche, on a basal membrane, houses different cell populations, such as fibroblasts, melanocytes, immune cells, vascular endothelial cells, and mesenchymal stem cells (MSCs), among others [7, 8]. However, due to its cellular complexity and heterogeneity, there are still many unknowns about the roles of LSCs and their secretory and proteomic profiles. Previous studies suggest that in the LSC niche, different substances with biological activity responsible for the proper functioning of the niche are released [7].

Although LSCs have been studied in humans [9, 10], in recent years their role in physiological and pathological conditions in veterinary medicine has begun to be evaluated [11, 12]. Although LSC transplantation is a therapeutic strategy in human medicine in cases of damage or insufficiency [5, 13], whose purpose is to restore the integrity of the corneal epithelium, such therapy is nearly unknown in dogs [14]. To date, neither the secretory profile of canine LSCs (cLSCs) nor their exosomes have been characterized. There are no studies on the interaction of cLSCs with other cell types located in its niche that play an important role in certain ocular pathologies. In this context, the formation of escar on the corneal surface is a frequent complication of corneal stromal healing in response to surgery, trauma, or infection, disturbing visual clarity. The cells responsible for the formation of scars are mitotically active fibroblasts originating from stromal keratocytes [15]. There is little literature on the role of LSCs in stromal healing [16].

The objectives of this study were (1) to characterize for the first time the soluble components and the proteomic profile of the cLSC secretome under standard culture conditions; and (2) to isolate, characterize, and determine the proteomic profile of the cLSC exosomes and compare these with those of the secretome. Finally, given the important role that fibroblasts play in the corneal stroma and in the sclerocorneal niche, we studied the in vitro effects of the secretome and exosomes of cLSCs.

## Materials and methods

All procedures with animals were performed by veterinarians in compliance with national and European legislation (Royal Decree RD1201/2005 and Directive 86/609/EEC of the EU, modified by 2003/65/EC, respectively) for the protection of animals used for research experimentation and other scientific purposes. Likewise, the protocols were approved by the Institutional Committee for the Care and Use of Animals of BIONAND (Andalusian Center for Nanomedicine and Biotechnology), Malaga, Spain, and written consent was obtained from all dog owners.

### Isolation, expansion and proliferation of cLSCs

Intact eyes of four young dogs free of any ocular pathology were extracted immediately after their slaughter, which was performed for reasons unrelated to this study. The eyes were immersed in Dulbecco's modified Eagle medium (DMEM) supplemented with 2.5 mM L-glutamine, 100 U/ml penicillin, 100 μg/mL streptomycin, and 1.25 μg/mL fungizone (Merck) and transported to our laboratory at 4°C, where they were processed within two hours postextraction.

On a sterile glass Petri dish, each eye was washed with saline, and with the aid of scalpel, scissors, and sterile forceps, fragments of approximately 0.5 x 0.5 cm of limbo-corneal tissue were obtained. After removal of iris debris and excess sclera, each explant was positioned with the epithelial surface upward in six-well plates with 3 ml of medium supplemented with 10% fetal bovine serum (FBS). This supplemented medium was composed of Dulbecco's modified Eagle medium (DMEM), 10% FBS, 2.5 mM L-glutamine, 100 U/ml penicillin, 100 μg/mL streptomycin, and 1.25 μg/mL fungizone (Merck) and will be referred to as supplemented medium.

The plates were incubated for three days under standard conditions (37°C, 95% humidity and 5% CO_2_), allowing cell migration from the explant to the wells. Then the explants were removed, each well was washed with 1 ml of phosphate-buffered saline (PBS), and 3 ml of supplemented medium was added to allow cell proliferation. Medium was refreshed every three days until the cells reached a confluence of 80%, at which time the subculture was performed. To do this, 0.5 ml of trypsin (Merck) was used per well, and the cells obtained were seeded in FT-75 flasks at a density of 8x10^5^ cells per bottle. Each donor was cultured individually and later frozen in 90% FBS and 10% dimethylsulfoxide (DMSO, Merck). All experiments were performed in cells of culture passage 2.

For the cLSC proliferation assay, we used the MTS colorimetric method (CellTiter 96 Aqueous One Solution Cell Proliferation Assay, Promega) following previously published protocols [17, 18]. For this, we plated 3000 cells/well in a 96-well plate with medium supplemented with 10% FBS. Medium was refreshed every two days, and readings were taken with a spectrophotometer (ELx800, Bio-Tek instruments) at a wavelength of 490 nm on days 1, 3, 6, 8, 10, and 13.

### Characterization of cLSCs

Using Western blot (WB), the expression of typical LSC markers ATP-binding cassette superfamily G member 2 (ABCG2), p63, anti-p40-deltaNp63, and vimentin was measured as described in the literature [4, 11, 12]. First, cellular proteins from cLSCs were extracted using RIPA buffer with protease inhibitors. This extract was kept on ice for 30 minutes, and the lysis mixture was centrifuged at 11,000 *g* and 4°C for 15 minutes. The supernatant was transferred to a new tube, and the protein concentration was determined using the bicinchoninic acid kit (BCA, SERVA).

For WB, 30 μg of protein was used, and the membranes were incubated with mouse anti-ABCG2 (Abcam, ab108312), mouse anti-p63 (Abcam, ab124762), anti-vimentin (Santa Cruz Biotechnology, sc-6260) and rabbit anti-deltaNp63 (Abcam, ab203826) at 1:1000 dilution. As a positive control for ABCG2, p63 and deltaNp63, a protein lysate from the HeLa cell line (Merck) was used, and in the case of vimentin, a protein lysate of human MSC from adipose tissue (hAd-MSC) was used as a positive control. Secondary anti-mouse (Merck) and anti-rabbit (Abcam) antibodies were used for detection by ECL (SERVA), and the results were visualized in the ChemiDocTM XRS + system (Bio-Rad).

### Obtaining the cLSC secretome

To obtain the cLSC secretome, 1.5 x 10^6^ cLSCs were seeded into FT-175 flasks with supplemented medium. Medium was refreshed twice a week. Once 80% confluence was reached, the medium was removed, and 20 ml supplemented medium per bottle was added. The cultures were maintained for 72 hours in the incubator, after which their conditioned medium was removed, passed through a 0.22-μm filter to remove cell debris, and frozen at -20°C. The cells of each flask were detached with trypsin and counted in a Neubauer chamber, and their viability was evaluated with trypan blue staining.

### cLSC secretory profile

For the quantification of cytokines and growth factors present in the cLSC secretome, an equal mixture of the media from the different donors was used. A commercial canine cytokine 11-plex assay (Thermo Fisher Scientific) was used, with which the concentration of 11 analytes was determined by Luminex technology: one chemokine (monocyte chemoattractant protein-1, MCP-1), cytokines (interleukin (IL)-2, IL-6, IL-8, IL-10, IL-12p40, tumor necrosis factor-α, interferon-γ), and growth factors (nerve growth factor-β, stem cell factor, vascular endothelial growth factor (VEGF)-A) [19]. The amounts obtained were normalized and are expressed in picograms per milliliter per million cells.

### Isolation and characterization of exosomes

To obtain the exosomes, 1 x 10^6^ cLSCs were seeded in each FT-175 flask with supplemented medium. Upon reaching 80% confluence, the flasks were washed with PBS, and medium supplemented with 10% FBS free of exosomes (ensured by prior ultracentrifugation at 100,000 *g* for 60 minutes at 4°C) was added. The medium was removed after 72 hours of incubation, the cells were counted in a Neubauer chamber, and their viability was evaluated with trypan blue.

The exosomes were isolated by ultracentrifugation as we have previously described [19]. In brief, the samples were centrifuged at 13,000 *g* and 4°C for 30 minutes to remove cell debris and microvesicles. Next, they were ultracentrifuged twice at 100,000 *g* for 60 minutes and 4°C using the ^70^Ti fixed-angle rotor in an Optima LE-80K ultracentrifuge (Beckman Coulter). The precipitate (exosomes) was resuspended in 100 μL of PBS to obtain a homogeneous solution. The exosomes were quantified using the BCA kit (Thermo Scientific) and stored at -20°C until use. Each sample was processed individually, and an equal mixture of each donor was performed to characterize the exosomes.

#### Transmission electron microscopy

A sample of 20 μL exosomes from cLSCs was resuspended in 0.1 M HEPES solution (pH 7.4) (Merck), and a drop was placed on a carbon-coated nickel grid (Aname) and allowed to dry overnight. Images of the samples were taken at different magnifications in a transmission electron microscope (TEM).

#### Determination of the size distribution and electronegativity

The exosomes were resuspended in 1 ml of PBS, and the size distribution and Z potential (electronegativity) were analyzed at 25°C using a Zetasizer Nano ZS (Malvern Instruments).

#### Western blot

The exosomes of cLSCs were characterized by WB for specific markers [20]. Briefly, 30 μg of exosomal proteins were used, and the membranes were incubated overnight with rabbit anti-ALIX (Abcam, ab186429), anti-TSG101 (Abcam, ab125011) and anti-calnexin (Cell Signaling Technology, 2679) antibodies at 1:1000 dilution. A protein lysate from human MSC from adipose tissue (hAd-MSC) was used as a positive control. Secondary anti-rabbit antibody (Abcam, ab6721) was used for detection by ECL (SERVA), and the results were visualized in the ChemiDoc XRS + system (Bio-Rad).

### Proteomic analysis of the secretome and exosomes of cLSCs

In the production of the secretome for proteomic analysis, the standard culture medium was replaced by DMEM without supplementation when the cell culture had reached 80% confluence. After 24 hours of incubation under standard conditions, the secretome was removed, filtered through a 0.22-μm, and frozen at -80°C. This secretome was lyophilized in the LyoQuest lyophilizer (TELSTAR) and reconstituted in a volume of 500 μl.

The proteomic profile was determined on an equal mixture of secretomes from different donors in the Proteomics Service of the SCAI of the University of Malaga, as described previously [19]. The tandem mass spectrometry data were compared against the SwissProt and NCBI nr databases according to the parameters described for “biological process” provided by the Gene Ontology resource.

### Activity of the secretomes and exosomes of cLSCs on fibroblasts

#### MTS proliferation assay

For the proliferation assay, 3000 canine fibroblasts (directly obtained from Cellider Biotech, catalogue number CFB001F) were seeded per well in a 96-well plate with the following conditions: control (100 μL medium supplemented with 10% FBS/well), secretome generated under standard conditions as described in section 3.3 (100 μL/well), and cLSC exosomes (25 μg/ml per well, added on days 1 and 5). The medium was refreshed every two days, and the proliferation was measured on days 1, 2, 5, 7, and 9 in the spectrophotometer (ELx800, Bio-Tek instruments) using the MTS assay (CellTiter 96 Aqueous One Solution Cell Proliferation Assay, Promega).

#### Wound-healing assay on canine fibroblasts in response to the cLSC secretome

Based on the results obtained above in the MTS assay, we evaluated the proliferation/migration capacity of fibroblasts using the wound healing assay. For this, canine fibroblasts were seeded at a density of 50,000 cells/cm^2^ in 24-well plates and kept in supplemented medium for 48 hours until they reached confluence. Using a pipette tip, a wound was generated in a straight line in the center of the well. Then, 500 μL of supplemented medium (C-) and the secretome from cLSCs (n = 4) were added.

The cultures were photographed with digital phase-contrast technology in an automated manner using an *Operetta High Content Screening* (HCS) *system* (Perkin Elmer) 0, 3, 7, 24, 48, and 120 hours after the application of the treatment.

The size of the open areas (areas without cells, corresponding to the scratch) was calculated by processing and analyzing the images with ImageJ software, applying the MRI *wound healing tool* (http://dev.mri.cnrs.fr/projects/imagejmacros/wiki/Wound_Healing_Tool). The open areas corresponding to time 0 were considered as 100%, and the rest of the areas were compared to this value for each replicate separately. Additionally, the shrinkage rate of the wound area was calculated for the period between 0 and 48 hours by calculating the slope in terms of mm^2^/day (Fig 6B). The average ± SD of each condition is shown (n = 4).

### Statistical analysis

Statistical analysis for the MTS proliferation assay was performed using one-way analysis of variance (ANOVA). In the case of the wound healing assay, the analysis used was two-way ANOVA for repeated measurements. Multiple comparisons between the different conditions were performed by a post hoc analysis using the Holm-Sidak method.

## Results

### Isolation and expansion of cLSCs

**Fig 1:**
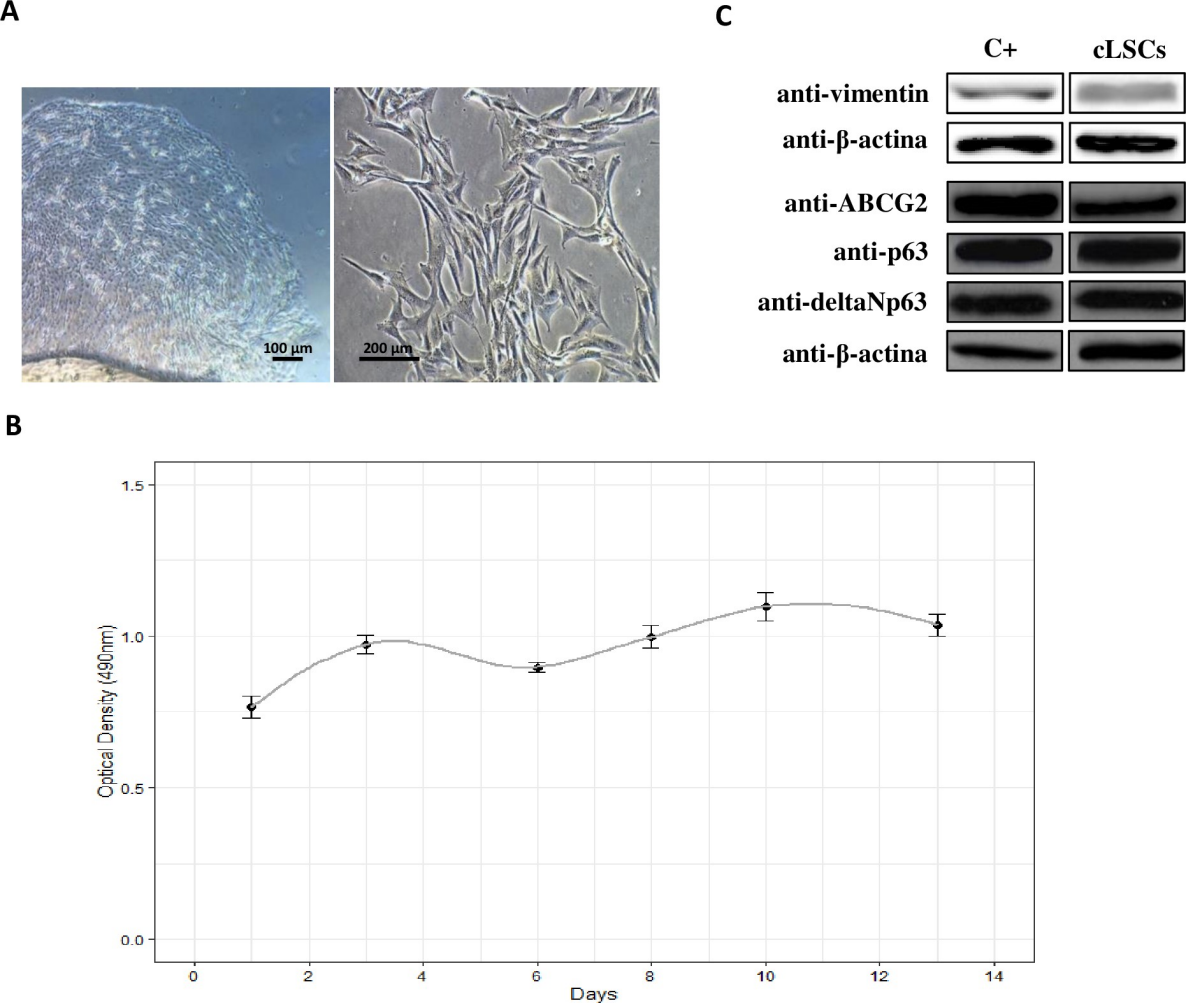
Isolation and characterization of cLSC. (A) Images were taken in culture passage 0 and 1, at 4x and 20x magnification respectively, under a bright field microscope. (B) Growth curve of cLSCs at culture passage 2 for 13 days by MTS assay. (C) Expression of ABCG2, p63, anti-p40-deltaNp63, and vimentin markers by WB. Positive control for ABCG2, p63 and deltaNp63 is a protein lysate from HeLa cell line; positive control for vimentin is a protein lysate of hAd-MSC.

The cLSCs began to migrate from the explant after 3 days of incubation, showing the characteristic morphology of this cell type (Fig 1A) and a proliferation that we could observe in the growth curve over 13 days at a culture passage 2 (Fig 1B).

### Western blot of the protein lysates of the cLSCs

Positive expression of the specific markers ABCG2, p63, anti-p40-deltaNp63, and vimentin in cLSCs was observed by WB in the protein lysates of these cells (Fig 1C).

### Characterization of the secretome composition by immunoassay

**Fig 2:**
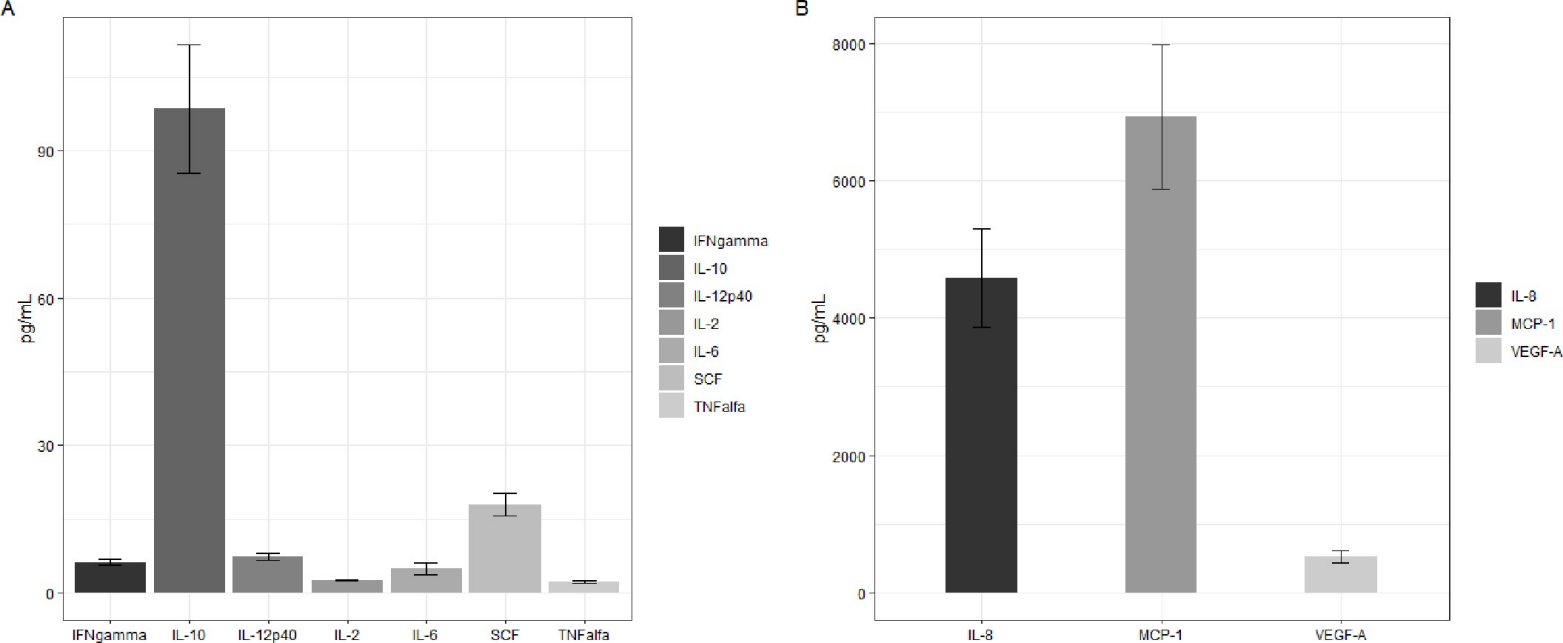
Profile of cytokines and growth factors present in cLSC secretome. (A) Analytes found in lower concentration. (B) Analytes more abundant in the secretome. Analyte concentrations are expressed as picograms/ml per million cells.

When the concentrations of the 11 analytes evaluated were quantified, we found higher amounts of MCP-1, IL-8, VEGF-A, and IL-10. The values obtained were normalized per million cells (Fig 2).

### Characterization of the exosomes of limbal cells

**Fig 3:**
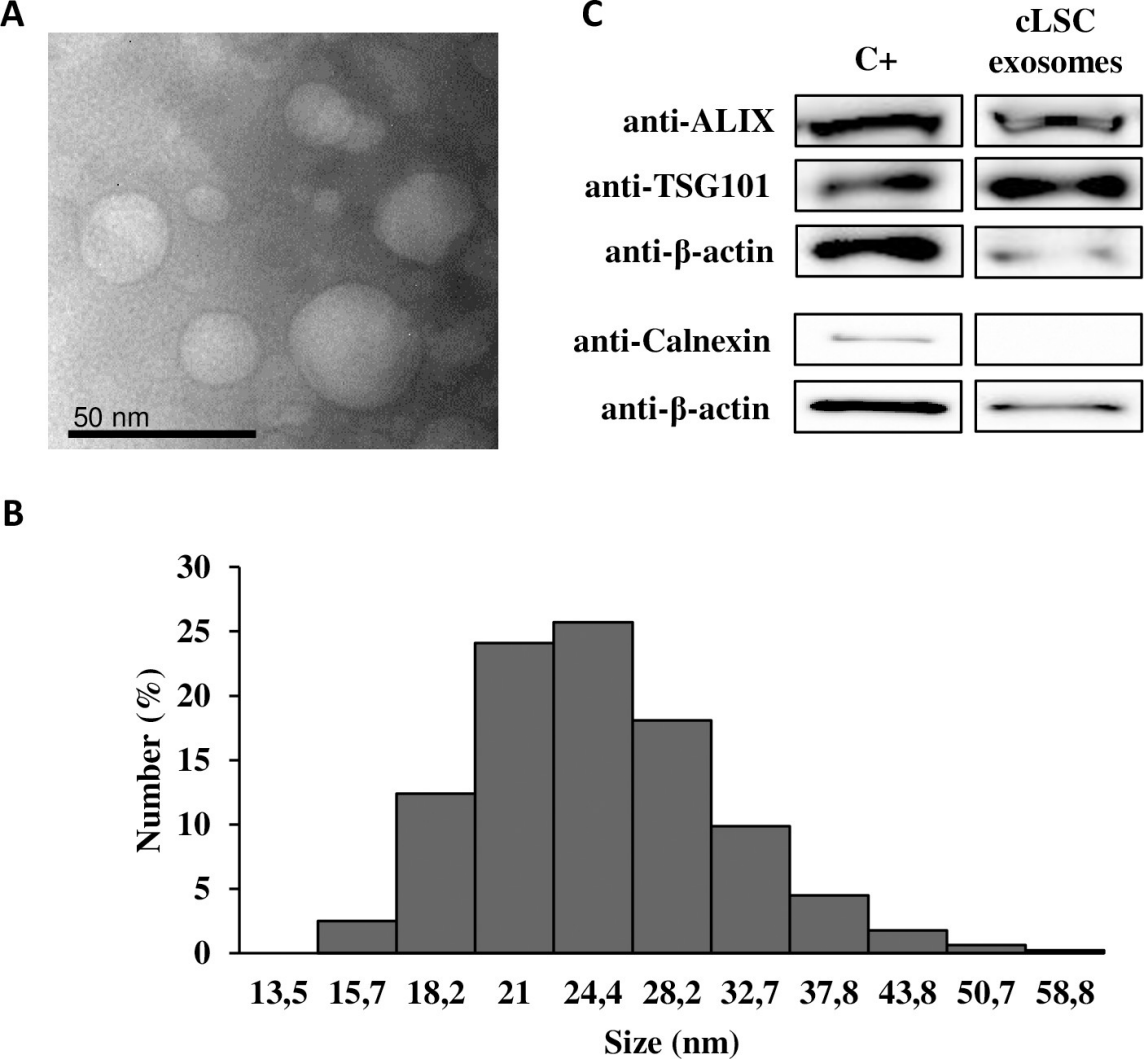
Exosome characterization. (A) Representative images of TEM. (B) Size distribution of exosomes using Zetasizer. (C) WB analysis of specific exosomal markers. A protein lysate of hAd-MSC was taken as positive control.

The exosomes of cLSCs examined by TEM showed a diameter less than 50 nm (Fig 3A). This was confirmed by dynamic light scattering to observe the size range of the isolated exosomes (Fig 3B). The detected Z potential was -11.8 ± 1.15 mV, and the polydispersity index (0.42) was indicative of a homogeneous sample. Fig 3C shows the positive expression of specific exosomal markers, such as ALIX and tumor susceptibility gene 101 (TSG101), and negative expression for calnexin.

### Proteomic analysis of the secretome and exosomes

**Fig 4:**
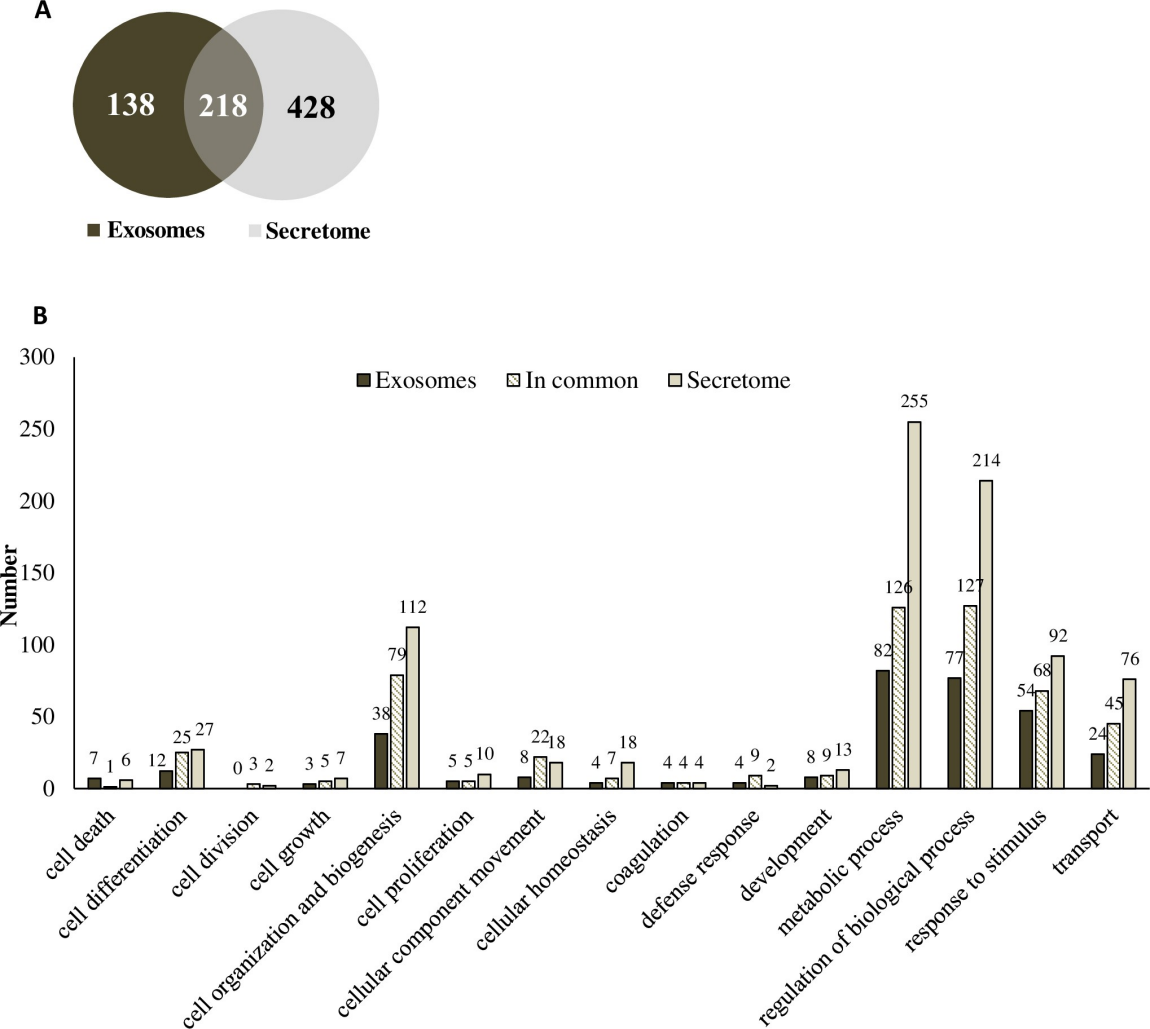
Proteomic analysis of cLSC secretome and exosomes. (A) Venn diagram showing the proteins present in exosomes (dark grey), secretome (light grey) and those common to both samples (overlapping colours). (B) Comparison of the biological processes involving the characterized proteins for exosomes (dark grey), secretome (light grey) and in common (striped) of cLSC according to Gene Ontology parameters.

The numbers of total proteins present in the secretome and exosomes of cLSCs were determined by mass spectrometry. For the bioinformatic analysis, the peptide database of the species *Canis lupus familiaris* was used. There were a total of 646 proteins in the secretome (light gray) and 356 in the exosomes (dark gray), and 218 proteins were shared in common between the secretome and exosomes (Fig 4A). These proteins were grouped according to the GO “biological processes” they are involved in according (Fig 4B). A specific list of all proteins material is indicated in the S1 and S2 Tables.

### Proliferation assays of canine fibroblasts under the influence of the limbal secretome

**Fig 5:**
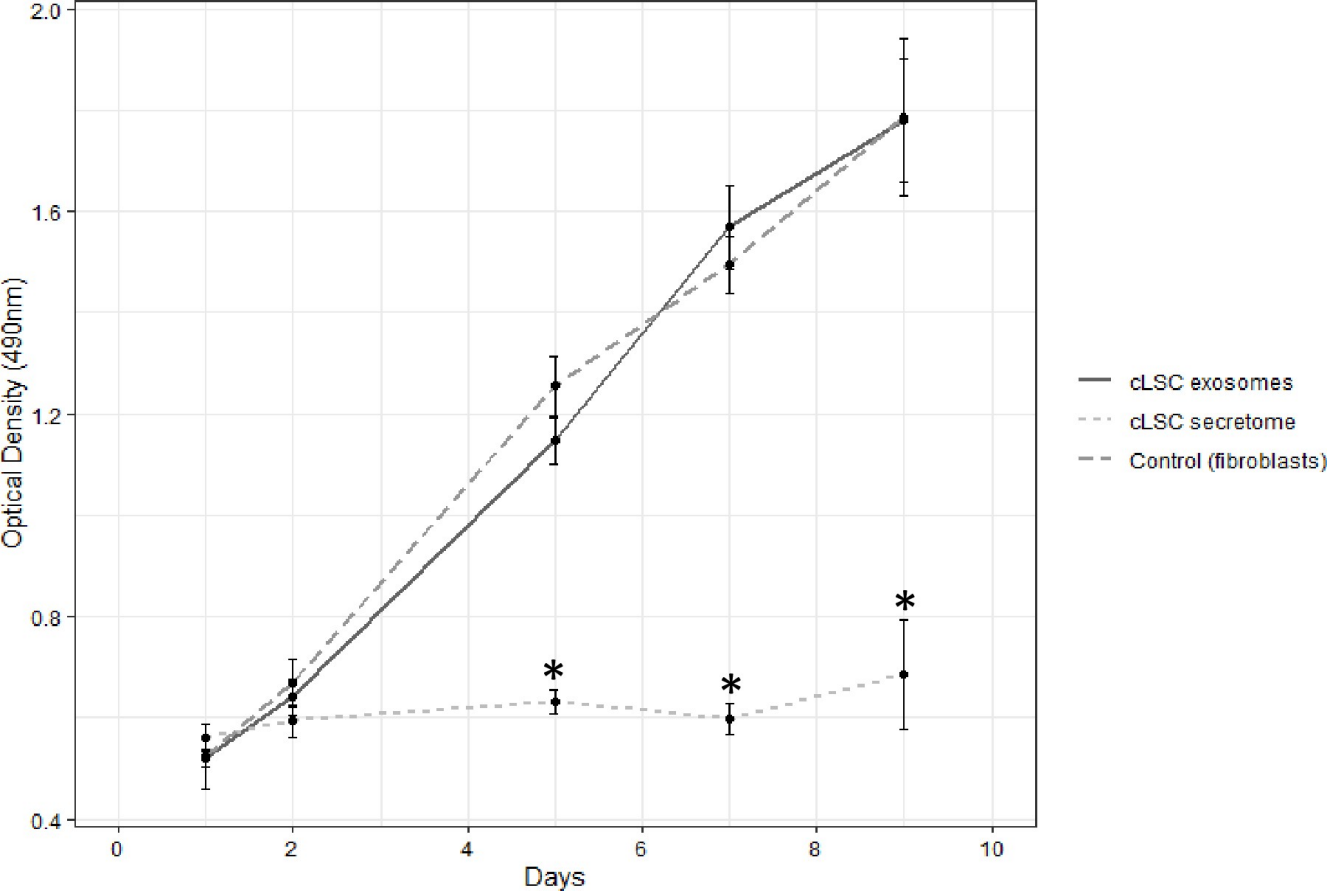
Proliferation assay. MTS showing the influence of exosomes (continuous dark grey curve) and secretome (discontinuous light grey curve) of cLSC on fibroblasts in culture (discontinuous dark grey curve). Statistically significant differences are shown on days 5, 7 and 9 *p<0.001 of the secretome versus exosomes and control condition.

The cLSC secretome showed inhibitory activity on the proliferation of canine fibroblasts, stopping this proliferation for the 9 days of the MTS assay (Fig 5). However, the exosomes of cLSCs did not show statistically significant differences from the control. It follows that the observed activity should be concentrated in the soluble fraction of the cLSC secretome and not in their exosomes.

#### Scratch-wound assay

**Fig 6:**
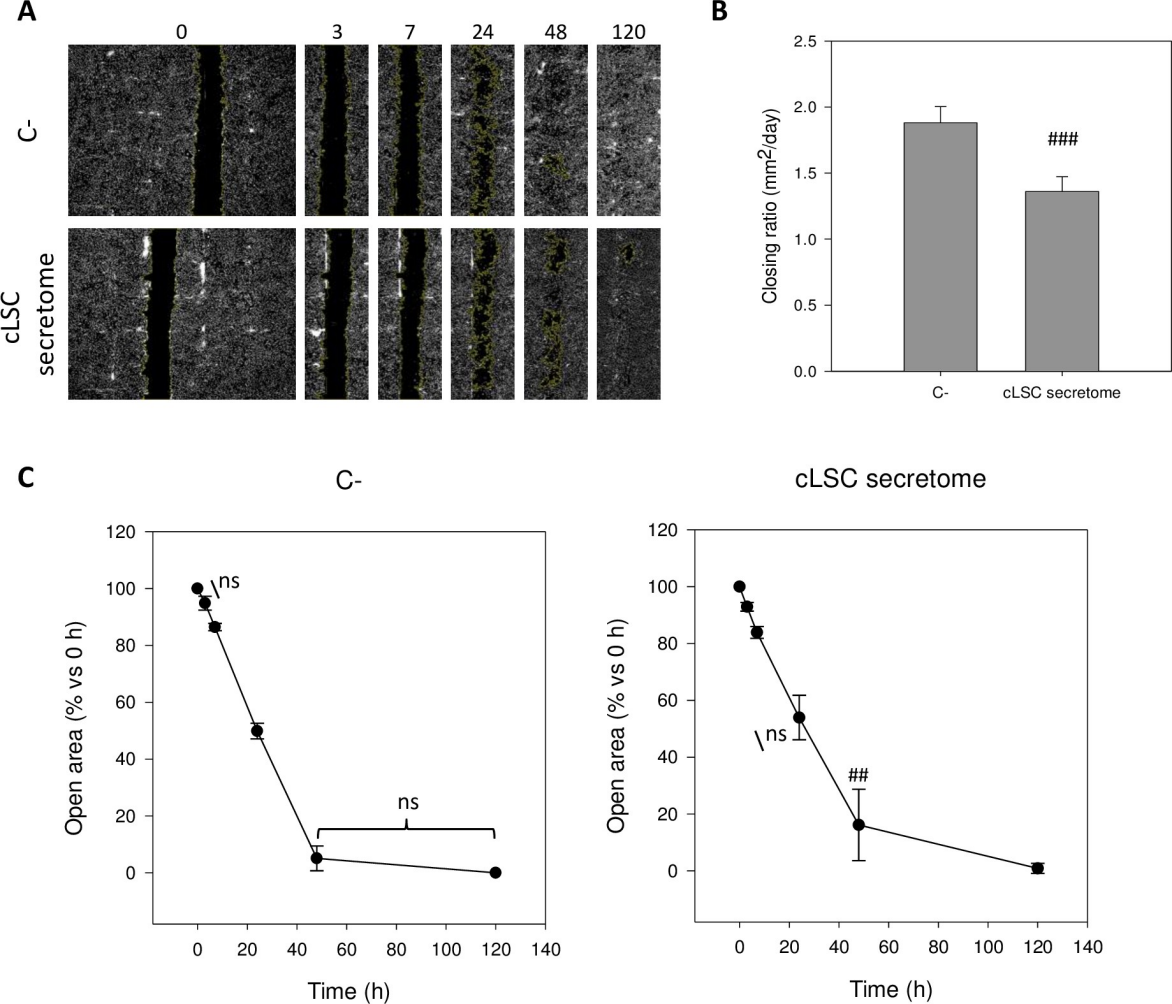
"Wound healing" test. (A) Example of representative replica photographs for each treatment and automatic detection of open areas. (B) Average rate of wound closure (in mm2/day) between 0 and 48h after treatment. (C) Evolution of the average open areas in time after the generation of the wound and the application of the treatment with control medium (C-) or cLSC secretome. Significant differences were detected between each time and the time immediately after, except for those indicated as not significant (ns). ##p<0.01; ###p<0.001; indicate significant differences between the marked condition and its C- homologous condition.

Fig 6 shows the effect of cLSC secretome treatment compared to the control medium on the migration of canine fibroblasts. At 48 hours posttreatment, the closure rate of the fibroblasts treated with control medium was 1.88 mm^2^/day, while treatment with the cLSC secretome significantly decreased the migration/growth rate to 1.36 mm^2^/day (p<0.001) (Fig 6B).

In the analysis of the percentage of open area, after 24 hours these areas were 4% higher in cultures treated with the secretome than in negative controls. This effect was statistically significant after 48 hours of treatment (Fig 6C). Fibroblasts treated with control medium migrated to decrease the open area to 5.07% of the initial area, while in cultures treated with the secretome, this area still made up on average 16.15%.

## Discussion

The regenerative processes of the cornea are complex mechanisms essential to ensure corneal transparency and visual function [16]. In this process, LSCs are responsible for the maintenance of the corneal epithelium through centripetal migration from their niche and their subsequent differentiation into corneal epithelial cells [21].

In the niche where they dwell, there are other cell types whose homeostasis depends on their interrelation [7, 22]. Within this specialized microenvironment, a great variety of signals are triggered through precise cellular and molecular mechanisms, which are just beginning to be elucidated [23], but knowledge of these signals is fundamental to understanding the processes of corneal repair/regeneration and to opening possibilities to new biological therapeutic strategies.

In this study, cLSCs were isolated from the sclerocorneal limbus by explant, and we proceeded to expand them in the laboratory without losing their undifferentiated nature. The cultures expressed markers such as ABCG2, p63, anti-p40-deltaNp63 and vimentin, related to typical characteristics of LSCs in different species [3, 24], including the canine species [12], confirming their origin from the basal limbal epithelium [25].

This study is the first to characterize soluble factors and the proteomic profile of the secretome and exosomes of cLSCs in culture. When analyzing their secretory profile, we quantified 11 soluble factors with different biological activities. Among them, MCP-1, IL-8, and VEGF were the most highly expressed, and IL-10 was also high. MCP-1, also known as CCL2, is a proinflammatory chemokine involved in the recruitment or migration of different cell types of the immune system and plays a direct mediating role in angiogenesis [26]. IL-8 or CXCL8 is a chemokine with the ability to induce chemotaxis and neutrophil degranulation, contributing to the elimination of pathogens, and it has a potent angiogenic capacity that promotes venous endothelial proliferation [27]. Through both of these cytokines, cLSCs could play important roles in inflammation and defense of the corneal surface against pathogens. When a pathogen comes into contact with the ocular surface, the innate immune system is activated, inducing a complex cascade of events, including the upregulation of proinflammatory cytokines involved in the repair of the ocular surface [28, 29].

VEGF is a signaling protein involved in the vasculogenesis and angiogenesis, in addition to manifesting vasodilator effects and increasing vascular permeability [30]. Through these actions, it plays an important role in the maintenance and nutrition of the abundant vascular structures of the sclerocorneal limbus [7, 31].

IL-10 is a pleiotropic cytokine with anti-inflammatory effects related to the induction of immune tolerance [32] and can intervene in the immune privilege enjoyed by the ocular surface. The ocular immune privilege confers immunological protection against the inflammatory response to organs, minimizing the risk of vision loss through strategies that modulate the innate and adaptive immune response and increase the immunological tolerance to different antigens [33, 34].

The proteomic profile of the cLSC secretome showed the existence of 646 different proteins, mainly assigned to functions such as transport, metabolism, regulation of different biological functions, cell differentiation, biogenesis, and response to stimuli.

Like other canine cell types [19], cLSCs produce exosomes, as shown by the characteristics that have been established for their identification, complying with the recommendation of the International Society of Extracellular Vesicles [20]. Exosomes are extracellular vesicles formed by a membrane unit that carries cargo that contains different bioactive molecules. They exert their effects by fusion with the target cells and transferring their cargo, playing a fundamental role in the intercommunication between the cells [35, 36]. Its role in corneal repair has been recently described in studies that evaluated exosomes of different cell types [37–39]. Through proteomic analysis of exosomes, we identified 356 proteins, mainly related to functions such as the regulation of different biological processes, metabolism, and response to stimuli. In addition, 218 proteins were shared between the proteomic profiles of the secretome and exosomes of cLSCs, among which we found proteins related to angiogenesis, growth, inflammation, metabolism, and cell signaling (S1 and S2 Tables).

The cargo of exosomes is specific to their cellular origin, but within the same species, a group of proteins essential for vesicular biogenesis, structure, and distribution is shared between exosomes from different cell types [35, 36]. To date, there is no specific description of common exosomal proteins of different origins in canine species. For this reason, when comparing cLSC proteomes with those of bone marrow and adipose tissue MSC exosomes previously published by our group [19], we discovered that they share nine proteins that have common functions related to biogenesis and cellular organization, regulation of biological processes, cellular metabolism, and transport (S1 and S2 Tables).

One of the most serious complications of corneal surface healing is the formation of eschar, whose persistence significantly compromises the transparency of the cornea [16, 40]. During its healing, the keratocytes of the corneal stroma differentiate into fibroblasts and myofibroblasts to induce the accumulation of extracellular matrix. This matrix is composed of altered glycosaminoglycans that contribute to reducing the transparency of scar tissue [41–43]. Although there is very little information on the role of LSCs in corneal stromal healing and their possible interaction with fibroblasts, we demonstrated in vitro that cLSCs were capable of inhibiting the proliferation and migration of fibroblasts. This inhibition occurred through the soluble factors of the secretome, as the application of their exosomes did not have an effect on proliferation or migration.

Considering all this, we conclude that LSCs, in the heterocellular complexity of the sclero-corneal niche, maintain a delicate balance in the regeneration of the ocular surface [39, 44]. LSCs in homeostatic conditions maintain a quiescent state [45] and play a central role in the maintenance of the niche through different molecules of their secretome, maintaining the vascular balance of the sclerocorneal limbus, defending against pathogens, and contributing to ocular immune privilege, in addition to exerting an inhibitory effect on the proliferation of corneal fibroblasts.

Recently, MSCs have been isolated and identified from the central and limbal stroma of the cornea in dogs [46], which opens new possibilities for future studies of the interconnection between both cell types to determine the contributions of cLSCs and MSCs in sclero-corneal physiological conditions and in ocular pathologies in canine species. LSC deficiency leads to corneal conjunctivization, whose progression leads to blindness [6]. This pathology has also been related to immune-mediated eye diseases of domestic species such as keratoconjunctivitis sicca and canine chronic superficial keratitis, as well as eosinophilic keratitis in cats and horses [2]. Our group has been a pioneer in describing the safety and efficacy of treatment with MSCs in both canine keratoconjunctivitis sicca [18] and feline eosinophilic keratitis [17]. We believe that the data provided in this study warrant new studies on the possible implantation of cLSCs alone or associated with MSCs in the treatment of such canine ocular pathologies.

Our study has certain limitations, such as the small number of donors, lack of specific markers, and a limited database of proteome studies for canine species.

All of the above leads us to propose that future objectives should focus on understanding the differentiation of LSCs during the regeneration/repair of the cornea and their relationship with other cell populations in order to better understand corneal pathophysiology. This knowledge will help to select the specific areas of the limbus or other parts of the cornea for the establishment of a cLSC culture for therapeutic purposes or as producers of secretomes and exosomes in vitro, which could be prepared in different pharmaceutical preparations and could be used as therapeutic elements.

## Supporting information

S1 Table Specific listing of cLSC exosome and secretome proteins according to the biological processes of GO. (PDF)

S2 Table Comparison of cLSC exosome proteins with bone marrow and adipose tissue MSC exosome proteins according to the biological processes of GO. (PDF)

S1 Raw images (PDF)
