# Supplementary material for: Characterization of the secretory profile and exosomes of limbal stem cells in the canine species
Source: PLoS One. 2020 Dec 29;15(12):e0244327. doi: 10.1371/journal.pone.0244327 (PMC7771867; doi:10.1371/journal.pone.0244327)
Supplement: S1 Table — (PDF) [file pone.0244327.s001.pdf]

List of specific proteins in cLSC exosomes							
**Number**	**Accession**	**Description**	**MW [kDa]**	**Score**	**Peptides**	**Coverage [%]**	**Biological Process**
1	XP_853995.2	EH domain-containing protein 3	60,9	65,23	15	37	Cell organization and biogenesis<br>Metabolic process<br>Regulation of biological process<br>Transport
2	XP_534040.1	Integrin-linked protein kinase	51,4	47,287	11	27	Cell organization and biogenesis<br>Cellular component movement<br>Development<br>Metabolic process<br>Regulation of biological process<br>Response to stimulus
3	XP_005631565.1	EH domain-containing protein 1 isoform X1	63,5	42,099	9	23	Cell organization and biogenesis<br>Metabolic process<br>Regulation of biological process<br>Transport
4	XP_013976674.1	Exostosin-2	82,3	41,719	8	17	Metabolic process
5	XP_003639703.1	Fermitin family homolog 3	75,3	41,285	12	21	Cell organization and biogenesis<br>Metabolic process<br>Regulation of biological process<br>Transport
6	XP_005635298.2	Tubulin beta-1 chain isoform X1	65,6	41,017	7	8	Cell organization and biogenesis

7	XP_851830.1	Contactin-1 isoform X1	113,3	37,743	13	17	Cell organization and biogenesis<br>Regulation of biological process
8	XP_005622605.1	Coagulation factor V	251,7	37,743	12	6	Coagulation
9	XP_533869.2	Cartilage oligomeric matrix protein	82,4	36,505	7	10	Regulation of biological process
10	XP_005631883.1	Protocadherin Fat 4	541,7	30,423	9	2	Cell differentiation<br>Cell organization and biogenesis<br>Cell proliferation<br>Regulation of biological process<br>Response to stimulus
11	XP_534989.4	Carboxypeptidase N catalytic chain isoform X1	50,8	29,097	4	12	Metabolic process
12	XP_005625819.2	Beta-parvin isoform X1	40,5	25,063	6	21	Cell organization and biogenesis<br>Regulation of biological process
13	XP_005630653.1	Apolipoprotein B-100	518	24,681	10	2	Transport
14	XP_539145.2	Exostosin-1	86,2	24,351	12	16	Cellular component movement<br>Metabolic process
15	XP_537942.2	Pancreatic alpha-amylase	57,7	23,451	4	10	Metabolic process
16	XP_003433491.1	Heparin cofactor 2	56,6	23,357	4	5	Regulation of biological process

17	XP_022280741.1	Complement C5	183,6	22,485	4	2	Metabolic process<br>Regulation of biological process<br>Response to stimulus
18	XP_538698.2	Cytoplasmic aconitate hydratase	98,3	20,264	6	9	Metabolic process
19	XP_003639742.1	Prothrombin	70,3	19,917	4	6	Cellular homeostasis<br>Coagulation<br>Defense response<br>Metabolic process<br>Regulation of biological process<br>Response to stimulus
20	XP_543688.2	ADP-ribosylation factor 3	20,6	19,442	5	35	Regulation of biological process<br>Response to stimulus
21	NP_001229641.1	A disintegrin and metalloproteinase with thrombospondin motifs 13	162,8	19,347	4	4	Metabolic process<br>Regulation of biological process<br>Response to stimulus
22	XP_005634263.1	Programmed Cell death 6-interacting protein isoform x1	96,8	18,984	9	12	Cell death<br>Cell organization and biogenesis<br>Metabolic process<br>Regulation of biological process<br>Response to stimulus<br>Transport
23	AIC64116.1	Glutathione S-transferase alpha 3	25,6	18,692	3	17	Metabolic process

24	NP_001019807.1	Ferritin light chain	20,1	18,654	5	21	Cellular homeostasis<br>Transport
25	XP_003435244.2	C-type mannose receptor 2	166,3	18,52	6	5	Metabolic process
26	XP_005629425.1	Fibrinogen gamma chain isoform X1	50,3	18,042	7	8	Cell organization and biogenesis<br>Regulation of biological process<br>Response to stimulus
27	XP_853123.1	Glycogen phosphorylase, muscle form isoform X2	97,1	17,907	5	6	Metabolic process
28	XP_005638634.1	Neogenin isoform X2	162,2	17,763	6	5	Metabolic process<br>Regulation of biological process<br>Response to stimulus
29	XP_533794.2	Inter-alpha-trypsin inhibitor heavy chain H1 isoform X1	100,9	16,768	4	4	Metabolic process<br>Regulation of biological process
30	XP_022259885.1	Protocadherin Fat 1 isoform X1	508	16,702	10	2	Cell differentiation<br>Cell organization and biogenesis<br>Cell proliferation<br>Regulation of biological process<br>Response to stimulus
31	XP_545677.2	Fibromodulin	43,1	16,504	2	6	Cell organization and biogenesis

32	XP_535142.3	Neuropilin-1 isoform X2	103,3	16,398	6	10	Cell differentiation<br>Cell growth<br>Cell organization and biogenesis<br>Cellular component movement<br>Development<br>Regulation of biological process<br>Response to stimulus<br>Transport
33	XP_022279152.1	14-3-3 protein epsilon isoform X4	30,3	15,665	6	27	Metabolic process<br>Regulation of biological process
34	NP_001003080.1	Ferritin heavy chain	21,3	15,532	4	22	Cellular homeostasis<br>Metabolic process<br>Regulation of biological process<br>Response to stimulus<br>Transport
35	XP_005616051.1	Asporin	42,4	15,459	5	18	Regulation of biological process<br>Response to stimulus
36	XP_536606.4	Beta-enolase isoform X1	47,8	15,235	2	7	Metabolic process<br>Regulation of biological process<br>Response to stimulus
37	XP_536832.2	Argininosuccinate lyase isoform X1	51,8	14,779	6	14	Metabolic process<br>Regulation of biological process<br>Response to stimulus

38	XP_022263056.1	Neurobeachin-like protein 2	298,7	14,695	6	3	Regulation of biological process
39	XP_022283816.1	Nucleosome assembly protein 1-like 1 isoform X1	45,3	14,531	3	12	Cell organization and biogenesis
40	XP_849464.1	Reelin isoform X1	389,3	14,164	9	3	Cell differentiation<br>Development<br>Regulation of biological process<br>Metabolic process<br>Response to stimulus<br>Transport
41	XP_005616668.1	Alpha-actinin-4 isoform X2	107,3	14,128	5	6	Cell organization and biogenesis<br>Metabolic process<br>Regulation of biological process<br>Transport
42	XP_005634613.1	Ceruloplasmin isoform X1	125,8	13,926	4	5	Metabolic process
43	XP_005619117.1	Adenosine kinase isoform X1	43,6	13,916	3	9	Regulation of biological process
44	XP_005640309.2	Prolyl endopeptidase FAP isoform X1	87,7	13,627	5	7	Cell death<br>Cell proliferation<br>Cellular component movement<br>Metabolic process<br>Regulation of biological process

45	XP_532697.2	Fibrinogen alpha chain	96,6	13,517	5	4	Cell organization and biogenesis<br>Coagulation<br>Defense response<br>Metabolic process<br>Regulation of biological process<br>Response to stimulus
46	NP_001041561.1	Glycogen debranching enzyme	174,7	13,355	5	4	Metabolic process
47	XP_863066.1	Ras-related protein Rap-1A isoform X2	21	13,125	4	20	Regulation of biological process<br>Response to stimulus
48	XP_005640957.1	Immunoglobulin superfamily member 8 isoform X1	66,6	12,726	4	9	Development
49	XP_533222.5	Beta-1,4-glucuronyltransferase 1	47,1	12,575	5	10	Cellular component movement<br>Metabolic process
50	XP_022262525.1	Plexin domain-containing protein 2 isoform X1	62,4	11,943	3	6	Development<br>Metabolic process<br>Regulation of biological process
51	XP_022261462.1	Ubiquitin thioesterase OTUB1 isoform X1	32	11,871	2	10	Metabolic process
52	XP_013977023.2	Cytosolic 10-formyltetrahydrofolate dehydrogenase	61,1	11,568	5	9	Metabolic process
53	XP_005625292.1	Argininosuccinate synthase	46,6	11,495	5	13	Metabolic process<br>Regulation of biological process<br>Response to stimulus

54	XP_005642305.2	Inter-alpha-trypsin inhibitor heavy chain H4 isoform X1	105,3	11,365	3	3	Metabolic process
55	XP_538011.3	Regucalcin	33,2	10,897	3	13	Metabolic process<br>Regulation of biological process
56	XP_005639121.1	General vesicular Transport factor p115 isoform X1	108,6	10,824	4	6	Cell organization and biogenesis<br>Transport
57	P18466.1	DLA class I histocompatibility antigen, A9/A9 alpha chain	40,4	10,627	2	8	Response to stimulus
58	XP_022277174.1	Microtubule-associated protein RP/EB family member 2 isoform X1	40,1	10,595	3	13	Regulation of biological process
59	NP_001136142.1	Erythrocyte band 7 integral membrane protein	31,2	10,548	3	15	Regulation of biological process
60	XP_851154.1	Carboxypeptidase B2	49,1	9,965	3	4	Metabolic process<br>Regulation of biological process
61	XP_536229.2	Dihydropteridine reductase	25,6	9,553	2	14	Metabolic process
62	XP_005637046.1	Dynamin-1-like protein isoform x1	83,4	9,274	4	6	Cell death<br>Cell organization and biogenesis<br>Regulation of biological process
63	XP_013966836.1	Thyroxine-binding globulin isoform X1	49,4	9,243	2	6	Regulation of biological process<br>Transport

64	XP_536313.2	Betaine-homocysteine S-methyltransferase 1 isoform X1	45,1	9,112	3	12	Metabolic process
65	NP_001003104.1	Galactokinase	42,1	9,066	4	9	Metabolic process
66	NP_001041495.2	Myocilin precursor	54,2	8,997	3	9	Cell differentiation<br>Cell organization and biogenesis<br>Regulation of biological process<br>Response to stimulus
67	XP_013968399.1	S-adenosylmethionine synthase	43,5	8,806	3	10	Cell organization and biogenesis<br>Metabolic process
68	XP_850088.2	Heat shock 70 kDa protein 13	51,9	8,684	4	9	Cell organization and biogenesis
69	XP_533187.3	26S proteasome regulatory subunit 6A	47,3	8,672	3	9	Metabolic process<br>Response to stimulus
70	XP_022260079.1	Prostaglandin F2 receptor negative regulator, partial	96,8	8,66	6	7	Metabolic process
71	XP_005637230.1	Pregnancy zone protein-like isoform X1	166,5	8,596	3	2	Development<br>Regulation of biological process
72	XP_005619392.1	Integrin alpha-1	130,4	8,587	4	3	Regulation of biological process<br>Response to stimulus
73	XP_548795.1	Alpha-1,3-mannosyl-glycoprotein 2-beta-N-acetylglucosaminyltransferase	51,6	8,549	3	9	Metabolic process

74	XP_003432942.1	Filamin-B isoform X1	281	8,486	6	2	Cell differentiation<br>Cell organization and biogenesis
75	XP_851917.1	Programmed Cell death protein 6 isoform X1	21,7	8,313	3	17	Cell death<br>Cell organization and biogenesis<br>Metabolic process<br>Regulation of biological process<br>Response to stimulus<br>Transport
76	XP_022272289.1	Alpha-mannosidase 2	125,3	8,24	3	3	Metabolic process
77	XP_013973206.2	Transforming growth factor-beta-induced protein ig-h3	84,6	8,196	6	7	Cell death<br>Cell differentiation<br>Cell growth<br>Cell organization and biogenesis<br>Cell proliferation<br>Cellular component movement<br>Metabolic process<br>Regulation of biological process<br>Response to stimulus<br>Transport
78	XP_854623.4	C-type lectin domain family 11 member A	35,7	8,109	4	15	Regulation of biological process
79	NP_001157926.1	Glutathione peroxidase 3 precursor	25,4	8,07	4	13	Metabolic process<br>Response to stimulus

80	XP_003640024.1	Matrix Gla protein	12,2	8,051	2	23	Cell differentiation<br>Development<br>Regulation of biological process<br>Response to stimulus
81	XP_536861.2	Guanine nucleotide-binding protein G(I)/G(S)/G(T) subunit beta-2	37,3	7,729	3	9	Regulation of biological process<br>Response to stimulus
82	NP_001003386.2	Keratin, type II cytoskeletal 2 epidermal	64,7	7,667	3	5	Cell organization and biogenesis<br>Cell proliferation<br>Cellular component movement<br>Metabolic process
83	XP_537434.1	Alpha-1,6-mannosyl-glycoprotein 2-beta-N-acetylglucosaminyltransferase	51,3	7,651	3	7	Metabolic process
84	XP_005626121.2	3-hydroxyanthranilate 3,4-dioxygenase	28,5	7,511	2	10	Metabolic process
85	XP_003432928.1	cAMP-dependent protein kinase type II-alpha regulatory subunit isoform X1	45,1	7,378	3	9	Regulation of biological process
86	AAB30434.1	Albumin, partial	30	7,351	2	11	Cell communication<br>Regulation of biological process<br>Response to stimulus<br>Transport
87	XP_003639307.1	Unconventional myosin-Ic isoform X1	121,9	7,255	4	4	Cell organization and biogenesis<br>Regulation of biological process<br>Response to stimulus<br>Transport

88	XP_022270009.1	Guanine nucleotide-binding protein subunit beta-4	37,6	7,232	3	9	Regulation of biological process<br>Response to stimulus
89	XP_533964.2	Basigin isoform X2	29,3	6,945	3	12	Cell differentiation<br>Development
90	XP_538701.5	Beta-1,4-galactosyltransferase 1	44,3	6,87	2	5	Metabolic process
91	XP_022280031.1	Glutamine--fructose-6-phosphate aminotransferase [isomerizing] 1	78,1	6,566	4	6	Metabolic process<br>Regulation of biological process<br>Response to stimulus
92	XP_022281366.1	Fructose-bisphosphate aldolase B isoform X1	40,2	6,531	3	9	Metabolic process
93	XP_005620276.1	Angiopoietin-related protein 3	53,8	6,529	3	6	Metabolic process<br>Regulation of biological process<br>Response to stimulus
94	XP_534027.2	Ribonucleoside-diphosphate reductase large subunit	90,2	6,468	3	3	Metabolic process
95	XP_013965764.1	Apolipoprotein D	23,2	6,26	3	13	Cell organization and biogenesis<br>Metabolic process<br>Regulation of biological process<br>Response to stimulus<br>Transport

96	XP_851244.1	Collectin-10 isoform X1	30,4	6,183	3	8	Response to stimulus
97	XP_546946.2	Acetylcholinesterase	67,3	6,126	3	6	Metabolic process
98	XP_542373.3	Hemoglobin subunit epsilon-2	16,4	6,088	2	13	Transport
99	XP_005615370.1	Cytosolic non-specific dipeptidase	52,6	6,064	2	6	Metabolic process
100	XP_022269621.1	Homogentisate 1,2-dioxygenase isoform X1	51,6	5,989	2	4	Metabolic process
101	XP_544572.4	Mannan-binding lectin serine protease 2	74,9	5,876	2	4	Defense response<br>Metabolic process<br>Regulation of biological process<br>Response to stimulus
102	XP_538538.2	Prenylcysteine oxidase 1	56,7	5,471	2	5	Metabolic process
103	XP_540242.1	Olfactomedin-like protein 3	46,2	5,466	3	7	Metabolic process
104	XP_853422.1	4-hydroxyphenylpyruvate dioxygenase	44,8	5,453	2	6	Metabolic process
105	XP_005629424.1	Fibrinogen beta chain	56,3	5,423	2	4	Cell organization and biogenesis<br>Coagulation<br>Defense response<br>Metabolic process<br>Regulation of biological process<br>Response to stimulus

106	NP_001239086.1	Proteasome subunit beta type-3	23	5,404	2	15	Metabolic process
107	XP_539652.3	Tyrosine-protein kinase receptor Tie-1	124,8	5,37	3	2	Cell organization and biogenesis<br>Metabolic process<br>Regulation of biological process<br>Response to stimulus
108	XP_022280917.1	Sorting nexin-2 isoform X1	46,6	5,297	3	9	Cell organization and biogenesis<br>Regulation of biological process<br>Response to stimulus;Transport
109	XP_022273570.1	Hydroxymethylglutaryl-CoA synthase, cytoplasmic isoform X1	61,6	5,219	2	4	Metabolic process
110	XP_547069.3	Coronin-1A	51	4,975	3	6	Cell organization and biogenesis<br>Cellular component movement<br>Regulation of biological process<br>Response to stimulus<br>Transport
111	XP_850996.1	Haptoglobin-like	38,4	4,966	3	7	Metabolic process<br>Regulation of biological process<br>Response to stimulus
112	XP_857475.2	Syntenin-1 isoform X1	32,4	4,93	2	10	Cell organization and biogenesis<br>Regulation of biological process<br>Response to stimulus

113	XP_022260186.1	Matrilin-3	52,4	4,894	2	6	Cell growth<br>Response to stimulus
114	NP_001003277.2	Ras-related protein rab-10	22,5	4,884	2	12	Cell differentiation<br>Cell organization and biogenesis<br>Regulation of biological process<br>Response to stimulus<br>Transport
115	XP_022259505.1	Bisphosphoglycerate mutase	30	4,862	2	9	Metabolic process<br>Regulation of biological process
116	XP_022269028.1	Formimidoyltransferase-cyclodeaminase isoform X3	63,2	4,857	2	4	Metabolic process
117	XP_005629167.1	Receptor-type tyrosine-protein phosphatase F isoform X1	213,5	4,849	3	2	Metabolic process
118	BAB20764.1	Catalase	59,8	4,451	2	4	Metabolic process<br>Regulation of biological process<br>Response to stimulus
119	XP_533617.1	Ras-related protein R-Ras	23,6	4,429	3	15	Cell differentiation<br>Regulation of biological process<br>Response to stimulus
120	XP_005638551.1	Ras-related protein Rab-8B	23,8	4,276	2	9	Regulation of biological process<br>Response to stimulus

121	XP_022276058.1	Glutathione S-transferase Mu 3 isoform X1	33	4,171	2	6	Metabolic process
122	NP_001003263.1	Guanine nucleotide-binding protein G(s) subunit alpha	45,6	4,101	2	7	Regulation of biological process<br>Response to stimulus
123	XP_536694.2	Complement component C8 beta chain	67	4,035	2	5	Response to stimulus
124	XP_848306.1	Fructose-1,6-bisphosphatase 1 isoform X1	36,8	3,96	2	8	Cell organization and biogenesis<br>Metabolic process<br>Regulation of biological process<br>Response to stimulus
125	XP_538392.4	Ras-related C3 botulinum toxin substrate 2	21,4	3,736	2	11	Cell organization and biogenesis<br>Regulation of biological process<br>Response to stimulus
126	XP_535960.2	Cytochrome b reductase 1	30,9	3,421	2	10	Metabolic process<br>Regulation of biological process<br>Transport
127	XP_022273264.1	Oncoprotein-induced transcript 3 protein isoform X1	60,3	3,256	2	4	Cellular homeostasis<br>Regulation of biological process
128	XP_851185.1	Protein NDRG2 isoform X1	40,7	3,097	2	7	Regulation of biological process<br>Response to stimulus
129	NP_001273906.1	Retinal dehydrogenase 1	54,9	3,029	2	4	Metabolic process

130	XP_548138.2	26S proteasome non-ATPase regulatory subunit 3	60,9	2,824	2	4	Metabolic process<br>Regulation of biological process
131	XP_003639883.1	Sorting nexin-5 isoform X1	46,7	2,744	2	5	Cell organization and biogenesis<br>Transport
132	XP_534923.1	Peptidyl-prolyl cis-trans isomerase FKBP4 isoform X1	51,5	2,639	2	4	Cell organization and biogenesis<br>Metabolic process<br>Regulation of biological process<br>Response to stimulus
133	NP_001240671.1	Keratin, type I cytoskeletal 19	43,9	2,544	2	4	Cell differentiation<br>Cell organization and biogenesis<br>Regulation of biological process<br>Response to stimulus
134	XP_851427.1	Xylulose kinase isoform X1	58,1	2,52	2	4	Metabolic process
135	XP_852675.1	Carbonyl reductase [NADPH] 1	30,6	2,51	2	5	Metabolic process
136	XP_533447.4	Protein-L-isoaspartate(D-aspartate) O-methyltransferase isoform X1	30,3	2,471	2	8	Metabolic process
137	XP_022272464.1	Dihydrofolate reductase isoform X1	17,7	2,359	2	10	Metabolic process<br>Regulation of biological process<br>Transport
138	XP_854204.2	CD276 antigen	57	2,241	2	5	Regulation of biological process

List of specific proteins in cLSC secretome							
**Number**	**Accession**	**Description**	**MW [kDa]**	**Score**	**Peptides**	**Coverage [%]**	**Biological Process**
1	XP_005628115.1	Plectin isoform X10	518,6	432,987	130	31	Cell organization and biogenesis<br>Regulation of biological process
2	XP_853410.1	Alpha-actinin-4 isoform X3	104,9	407,254	53	57	Cell organization and biogenesis<br>Metabolic process<br>Regulation of biological process<br>Transport
3	XP_539385.1	Filamin-C isoform X1	290,2	330,894	68	31	Cell organization and biogenesis<br>Development
4	XP_541829.2	Filamin-B isoform X4	277,5	329,972	71	35	Cell differentiation<br>Cell organization and biogenesis
5	XP_022268508.1	Pyruvate kinase PKM isoform X2	64,5	238,224	30	47	Metabolic process
6	NP_001274080.1	Lamin	74,2	235,103	42	55	Cell organization and biogenesis<br>Regulation of biological process
7	NP_001003349.2	Actin, cytoplasmic 2	41,7	227,273	23	58	Cell organization and biogenesis<br>Cellular component movement<br>Regulation of biological process

8	XP_003432953.1	Tropomyosin alpha-4 chain isoform X3	28,5	158,773	27	65	Cell differentiation<br>Cell organization and biogenesis
9	XP_864981.1	Tropomyosin alpha-1 chain isoform X11	32,9	150,411	26	63	Cell differentiation<br>Cell organization and biogenesis
10	XP_852641.1	Tropomyosin alpha-4 chain isoform X1	32,7	133,466	23	53	Cell differentiation<br>Cell organization and biogenesis
11	XP_851009.1	Collagen alpha-1(III) chain	138,4	133,435	23	18	Cell organization and biogenesis<br>Coagulation<br>Development<br>Metabolic process<br>Regulation of biological process<br>Transport
12	XP_538720.2	Tropomyosin beta chain isoform X4	33	130,44	23	50	Cell differentiation<br>Cell organization and biogenesis
13	XP_005632479.1	Transketolase	63,1	125,749	21	37	Metabolic process<br>Regulation of biological process
14	XP_005638545.1	Tropomyosin alpha-1 chain isoform X12	32,7	116,086	21	67	Cell differentiation<br>Cell organization and biogenesis

15	XP_022279644.1	Collagen alpha-1(V) chain	203,9	110,555	18	11	Cell organization and biogenesis<br>Coagulation<br>Development<br>Metabolic process<br>Regulation of biological process<br>Transport
16	XP_005622310.1	Tropomyosin alpha-3 chain isoform X6	29	100,375	16	54	Cell differentiation<br>Cell organization and biogenesis
17	XP_022259500.1	Caldesmon isoform X1	85,9	99,727	21	37	Response to stimulus
18	XP_022276121.1	Collagen alpha-1(XI) chain isoform X4	181,7	92,671	15	13	Cell organization and biogenesis<br>Coagulation<br>Development<br>Metabolic process<br>Regulation of biological process<br>Transport
19	XP_005626978.1	Prostaglandin reductase 1	35,7	85,34	14	47	Metabolic process
20	XP_005624419.1	Pigment epithelium-derived factor isoform X1	46,5	81,935	13	35	Regulation of biological process
21	XP_542822.4	Procollagen-lysine,2-oxoglutarate 5-dioxygenase 2 isoform X1	87,6	80,981	17	22	Metabolic process<br>Response to stimulus
22	XP_005642097.1	Glucose-6-phosphate 1-dehydrogenase isoform X1	63,6	73,694	20	34	Metabolic process

23	XP_852626.1	Transitional endoplasmic reticulum ATPase isoform X1	89,2	73,129	19	24	Metabolic process<br>Response to stimulus
24	XP_022266958.1	Heterogeneous nuclear ribonucleoprotein A1	38,8	71,466	12	40	Metabolic process<br>Regulation of biological process
25	XP_005628594.1	Asparagine synthetase [glutamine-hydrolyzing]	64,4	70,076	19	40	Cell communication<br>Metabolic process<br>Regulation of biological process<br>Response to stimulus
26	NP_001003229.1	Biglycan precursor	41,5	63,662	11	34	Regulation of biological process<br>Response to stimulus
27	XP_005630547.1	Macrophage-capping protein isoform X1	38,8	61,284	9	36	Cell organization and biogenesis<br>Regulation of biological process
28	XP_540488.2	Protein disulfide-isomerase	57,4	59,18	19	33	Cellular homeostasis<br>Metabolic process<br>Regulation of biological process<br>Response to stimulus
29	XP_005640882.1	Bifunctional glutamate/proline--tRNA ligase isoform X1	170,6	58,793	19	16	Metabolic process
30	P38486.3	Galectin-3	30,3	58,267	9	28	Cell differentiation<br>Defense response<br>Metabolic process<br>Response to stimulus

31	XP_022274045.1	Radixin isoform X1	71	57,88	13	19	Cell organization and biogenesis<br>Regulation of biological process<br>Response to stimulus
32	XP_005618292.1	Nuclease-sensitive element-binding protein 1	36	57,236	8	43	Regulation of biological process
33	AAF67517.1	Matrix metalloproteinase-2, partial	70,9	56,068	17	27	Metabolic process
34	XP_003639538.1	Glycine--tRNA ligase isoform X1	83,6	53,849	16	22	Metabolic process
35	NP_001003179.1	Ribosome-binding protein 1	164,5	53,769	19	15	Metabolic process<br>Regulation of biological process<br>Response to stimulus<br>Transport
36	XP_022272229.1	Adipocyte enhancer-binding protein 1, partial	101,7	51,347	13	18	Metabolic process
37	XP_532436.3	Staphylococcal nuclease domain-containing protein 1	101,9	50,68	17	21	Cell differentiation<br>Metabolic process<br>Regulation of biological process
38	XP_854358.1	14-3-3 protein epsilon isoform X2	29,2	49,916	13	55	Metabolic process<br>Regulation of biological process
39	NP_001019809.1	Nucleoside diphosphate kinase b	17,4	49,125	8	53	Metabolic process<br>Regulation of biological process<br>Response to stimulus

40	NP_001116145.1	Thioredoxin reductase 1, cytoplasmic	71,5	48,628	13	24	Cellular homeostasis<br>Metabolic process<br>Regulation of biological process
41	XP_005638858.2	T-complex protein 1 subunit theta isoform X1	60,4	48,42	16	31	Metabolic process<br>Regulation of biological process<br>Transport
42	NP_001271371.1	Procollagen-lysine,2-oxoglutarate 5-dioxygenase 1 precursor	83,5	47,957	11	18	Metabolic process
43	XP_013969069.1	Myosin-10 isoform X1	232,5	47,159	10	5	Metabolic process
44	XP_851909.2	Septin-2	41,5	46,7	9	36	Regulation of biological process<br>Response to stimulus
45	XP_005639700.1	Coiled-coil domain-containing protein 80	92,1	45,956	11	16	Cell organization and biogenesis<br>Regulation of biological process
46	NP_001041481.1	Heat shock 70 kDa protein 4	94,3	45,88	13	17	Cell organization and biogenesis
47	XP_022277656.1	Protein Transport protein Sec23A isoform X1	88,5	45,367	9	15	Transport
48	XP_022283282.1	Septin-7 isoform X1	49,4	44,655	9	29	Regulation of biological process<br>Response to stimulus
49	XP_533723.2	Coatomer subunit gamma-1	97,7	44,282	11	15	Transport

50	XP_534069.3	Coatomer subunit beta	107,1	43,619	10	17	Transport
51	XP_532876.2	Protein disulfide-isomerase A6	48,3	42,055	7	25	Cellular homeostasis<br>Metabolic process<br>Regulation of biological process<br>Response to stimulus<br>Transport
52	XP_849335.1	Calumenin isoform X1	37,1	41,007	9	27	Metabolic process<br>Regulation of biological process<br>Response to stimulus
53	XP_852131.1	Eukaryotic translation initiation factor 3 subunit A isoform X1	162,9	40,159	13	12	Cell organization and biogenesis<br>Metabolic process<br>Regulation of biological process
54	XP_013970474.1	Sulfhydryl oxidase 1, partial	79,2	39,937	9	17	Cellular homeostasis<br>Metabolic process<br>Regulation of biological process
55	XP_022262693.1	Peroxiredoxin-2 isoform X1	26	39,684	8	50	Cellular homeostasis<br>Metabolic process<br>Regulation of biological process
56	XP_547813.2	ERO1-like protein alpha	54,3	39,322	10	22	Cell differentiation<br>Cell organization and biogenesis<br>Cellular homeostasis<br>Metabolic process<br>Regulation of biological process<br>Response to stimulus<br>Transport

57	XP_013965795.1	T-complex protein 1 subunit epsilon	59,6	38,824	9	18	Metabolic process<br>Regulation of biological process<br>Transport
58	XP_849060.3	Asparagine--tRNA ligase, cytoplasmic isoform X1	64,2	38,171	10	15	Metabolic process
59	XP_536894.4	Eukaryotic translation initiation factor 3 subunit B	89,1	37,925	8	17	Cell organization and biogenesis<br>Metabolic process<br>Regulation of biological process
60	XP_013972487.1	Spectrin alpha chain, non-erythrocytic 1 isoform X1	288,9	37,445	13	6	Cell organization and biogenesis<br>Metabolic process<br>Regulation of biological process<br>Transport
61	XP_022270850.1	Actin-related protein 2/3 complex subunit 2	33,8	36,37	9	24	Cell organization and biogenesis<br>Regulation of biological process
62	XP_022269683.1	F-actin-capping protein subunit beta isoform X3	34,4	36,104	5	21	Cell organization and biogenesis<br>Regulation of biological process
63	XP_005618600.1	Cytosol aminopeptidase	56,2	35,597	9	21	Metabolic process
64	XP_013966632.1	Coatomer subunit alpha, partial	137,7	35,592	13	11	Regulation of biological process<br>Transport
65	XP_022282119.1	Prolyl endopeptidase	72,9	35,547	10	18	Metabolic process<br>Regulation of biological process

66	NP_001243477.1	Heterogeneous nuclear ribonucleoprotein K	50,9	34,884	9	23	Metabolic process<br>Transport
67	XP_853102.1	Arginine--tRNA ligase, cytoplasmic	75,5	34,657	10	17	Metabolic process
68	NP_001238869.1	Eukaryotic translation initiation factor 3 subunit L	66,7	34,626	8	17	Cell organization and biogenesis<br>Metabolic process<br>Regulation of biological process
69	XP_542066.2	Interleukin enhancer-binding factor 3 isoform X1	96	34,492	11	14	Metabolic process
70	XP_853723.1	AP-2 complex subunit beta isoform X1	105,6	34,429	13	14	Transport
71	XP_858778.1	Calumenin isoform X2	37,1	34,33	8	27	Metabolic process<br>Regulation of biological process<br>Response to stimulus
72	XP_003639985.1	Gamma-enolase	47,2	34,075	6	20	Metabolic process<br>Regulation of biological process<br>Response to stimulus
73	XP_535555.3	1,4-alpha-glucan-branching enzyme	80,6	33,428	9	15	Metabolic process
74	XP_548076.1	Synaptic vesicle membrane protein VAT-1 homolog	42,5	33,379	8	33	Metabolic process
75	XP_536856.2	Procollagen-lysine,2-oxoglutarate 5-dioxygenase 3	84,7	33,317	9	14	Cell organization and biogenesis<br>Metabolic process

76	XP_005634562.1	Coatomer subunit beta' isoform X1	102,3	33,258	8	11	Transport
77	XP_853617.3	NAD(P)H dehydrogenase [quinone] 1	30,6	33,174	8	25	Metabolic process
78	XP_005641149.2	Anosmin-1 isoform X1	75,2	32,67	10	23	Regulation of biological process
79	XP_022261215.1	AP-2 complex subunit alpha-2 isoform X1	103,9	32,439	12	13	Transport
80	XP_005624202.1	Fatty acid synthase	268,9	32,238	10	5	Metabolic process
81	XP_005619329.1	Sparc	34,5	31,909	5	11	Regulation of biological process<br>Response to stimulus
82	XP_005623717.1	Latent-transforming growth factor beta-binding protein 2	195	31,433	10	8	Cell death<br>Cell differentiation<br>Cell growth<br>Cell organization and biogenesis<br>Cell proliferation<br>Cellular component movement<br>Metabolic process<br>Regulation of biological process<br>Response to stimulus<br>Transport
83	XP_535324.4	Tyrosine--tRNA ligase, cytoplasmic isoform X1	63	31,045	11	21	Metabolic process
84	XP_541506.2	Nucleobindin-1	53,1	30,56	9	24	Regulation of biological process

85	XP_850270.1	Insulin-like growth factor-binding protein 7	28,9	30,53	6	25	Regulation of biological process
86	XP_005628702.1	Heterogeneous nuclear ribonucleoproteins A2/B1 isoform X1	37,4	30,248	9	40	Metabolic process;Transport
87	XP_848839.1	Myosin regulatory light polypeptide 9 isoform X1	20,5	29,502	8	39	Regulation of biological process
88	XP_022271459.1	Peroxiredoxin-4 isoform x1	30,9	29,298	6	22	Cellular homeostasis<br>Metabolic process<br>Regulation of biological process
89	XP_534215.2	Programmed Cell death 6-interacting protein isoform X2	96,1	28,77	10	14	Cell organization and biogenesis<br>Regulation of biological process
90	NP_001301047.1	Annexin A5	35,9	28,05	9	29	Regulation of biological process<br>Response to stimulus
91	XP_548098.3	Peptidyl-prolyl cis-trans isomerase FKBP10	64,3	27,954	9	17	Cell organization and biogenesis<br>Metabolic process
92	XP_531844.1	Malate dehydrogenase, cytoplasmic	36,4	27,384	5	20	Metabolic process
93	XP_536872.1	Actin-related protein 2/3 complex subunit 1B	41	27,296	9	28	Cell organization and biogenesis<br>Regulation of biological process
94	XP_542053.1	Calponin-1 isoform X2	33,2	27,23	7	20	Cell organization and biogenesis

95	XP_022276360.1	T-complex protein 1 subunit zeta	63,6	27,101	11	16	Metabolic process<br>Regulation of biological process<br>Transport
96	XP_005623483.1	Kinectin isoform X8	156,5	26,725	13	10	Transport
97	XP_535269.4	Microtubule-associated protein 1B isoform X1	270,2	26,342	9	4	Regulation of biological process
98	XP_849674.1	Dynactin subunit 2 isoform X1	45	26,191	7	26	Cell organization and biogenesis<br>Cellular component movement<br>Regulation of biological process
99	XP_022268860.1	Collagen alpha-2(VI) chain isoform X2	82,3	26,077	8	11	Cell organization and biogenesis<br>Regulation of biological process<br>Response to stimulus
100	XP_861533.1	Lysine--tRNA ligase isoform X1	71,5	26,054	9	12	Metabolic process<br>Regulation of biological process<br>Response to stimulus
101	XP_536923.2	Eukaryotic translation initiation factor 3 subunit C	105,4	25,741	9	12	Cell organization and biogenesis<br>Metabolic process<br>Regulation of biological process
102	XP_856708.1	Polyadenylate-binding protein 1 isoform X1	70,6	25,432	7	13	Regulation of biological process
103	XP_852389.1	Peptidyl-prolyl cis-trans isomerase B	23,8	25,09	10	40	Metabolic process

104	XP_852707.1	Actin-related protein 2 isoform X1	45,3	24,832	6	19	Cell organization and biogenesis<br>Regulation of biological process
105	XP_005639179.1	Protein Transport protein Sec31A isoform X1	137,5	24,654	7	8	Transport
106	XP_013965972.1	Histone H1.2-like	25,7	24,611	5	14	Cell organization and biogenesis
107	XP_022260851.1	Uridine phosphorylase 1	45,4	24,465	5	15	Metabolic process
108	XP_022282560.1	Elongation factor 1-delta isoform X1	69,6	24,413	5	11	Metabolic process<br>Regulation of biological process<br>Response to stimulus
109	XP_003639324.1	Caveolae-associated protein 1	43,4	24,22	3	12	Metabolic process<br>Regulation of biological process
110	XP_543244.3	Lysyl oxidase homolog 2	86,5	24,184	10	11	Metabolic process
111	XP_003434465.1	Versican core protein isoform X1	369,3	24,073	8	3	Cell differentiation
112	XP_866214.1	Serine-tRNA ligase, cytoplasmic	58,5	23,937	5	13	Metabolic process
113	XP_003639276.1	Proteasome subunit beta type-5	28,5	23,693	5	22	Metabolic process<br>Response to stimulus

114	XP_022281426.1	Spliceosome RNA helicase DDX39B isoform X1	49,8	23,361	8	22	Cell organization and biogenesis<br>Metabolic process<br>Regulation of biological process<br>Response to stimulus<br>Transport
115	XP_533146.1	Transaldolase	37,5	23,345	7	26	Metabolic process
116	XP_867372.2	Ras GTPase-activating protein-binding protein 1	52	23,302	4	12	Transport
117	XP_003640149.1	COP9 signalosome complex subunit 4 isoform X1	46,2	23,192	6	19	Metabolic process
118	XP_531631.3	Myosin light polypeptide 6 isoform X1	17	23,071	6	40	Metabolic process
119	XP_539728.3	Leukotriene A-4 hydrolase	69,2	22,972	7	15	Metabolic process
120	XP_022280457.1	Spectrin beta chain, non-erythrocytic 1 isoform X1	274,3	22,959	7	3	Cell organization and biogenesis<br>Metabolic process<br>Regulation of biological process<br>Transport
121	NP_001274075.1	Major vault protein	99,1	22,667	9	12	Regulation of biological process<br>Response to stimulus
122	XP_005639374.1	Protein Transport protein Sec24D isoform X1	112,8	22,472	6	8	Transport
123	XP_005633960.1	Translationally-controlled tumor protein	19,6	22,372	3	20	Cell differentiation

124	XP_854960.4	Stress-induced-phosphoprotein 1	67,2	22,029	8	14	Metabolic process
125	XP_536552.2	Coatomer subunit delta	57,2	21,858	7	12	Response to stimulus<br>Transport
126	XP_013971460.1	40S ribosomal protein SA-like	32,7	21,773	4	20	Metabolic process
127	XP_532223.3	Heterogeneous nuclear ribonucleoprotein Q isoform X5	69,6	21,542	6	13	Metabolic process<br>Transport
128	XP_005622256.1	Cysteine and glycine-rich protein 1	20,6	21,461	4	28	Metabolic process
129	XP_022280011.1	Probable ATP-dependent RNA helicase DDX17, partial	80,7	20,995	7	11	Cell organization and biogenesis<br>Metabolic process<br>Regulation of biological process
130	XP_852198.1	Ap-1 complex subunit beta-1 isoform x1	104,7	20,841	7	8	Transport
131	XP_537263.1	Interleukin enhancer-binding factor 2	43	20,825	6	21	Response to stimulus
132	XP_005617343.1	Histidine-tRNA ligase, cytoplasmic isoform X1	57,5	20,79	8	16	Metabolic process
133	NP_001003164.1	Alpha-centractin	42,6	20,764	3	13	Cell organization and biogenesis<br>Cellular component movement<br>Regulation of biological process

134	XP_022265718.1	Nucleolin isoform X1	78,7	20,631	7	10	Metabolic process<br>Regulation of biological process<br>Response to stimulus
135	NP_001239096.1	Glutathione S-transferase pi 1	23,5	20,583	2	16	Metabolic process
136	NP_001003228.1	Decorin precursor	40	20,266	6	19	Metabolic process<br>Regulation of biological process<br>Response to stimulus
137	XP_022281407.1	Thioredoxin-like isoform X1	20,6	20,236	2	14	Cellular homeostasis<br>Metabolic process<br>Regulation of biological process<br>Response to stimulus<br>Transport
138	XP_005624092.1	Soluble calcium-activated nucleotidase 1 isoform X1	94,3	20,179	6	10	Metabolic process
139	XP_022279604.1	Septin-9	76,9	20,081	6	8	Regulation of biological process<br>Response to stimulus
140	XP_533666.2	Flavin reductase (NADPH) isoform X1	22,1	19,932	4	28	Metabolic process
141	XP_022270186.1	Serpin B6	42,7	19,909	4	15	Regulation of biological process
142	P42929.1	Heat shock protein beta-1	22,9	19,851	4	25	Metabolic process<br>Regulation of biological process

143	XP_013967762.1	Cytoplasmic FMR1-interacting protein 1	145,5	19,819	9	8	Cell growth<br>Regulation of biological process<br>Response to stimulus
144	XP_536040.1	Elongation factor 1-beta	24,8	19,728	4	24	Metabolic process
145	XP_548841.3	Matrix-remodeling-associated protein 5	310,1	19,618	10	4	Response to stimulus
146	XP_532813.2	Glutathione reductase, mitochondrial isoform X1	56,1	19,505	5	15	Cellular homeostasis<br>Metabolic process<br>Regulation of biological process
147	XP_854646.1	Calponin-2	33,4	19,377	4	16	Cell organization and biogenesis<br>Regulation of biological process<br>Response to stimulus
148	XP_005622749.1	Hepatoma-derived growth factor	23	19,312	5	25	Metabolic process<br>Regulation of biological process<br>Response to stimulus
149	XP_005631736.1	Triokinase/FMN cyclase isoform X1	59,4	19,274	5	13	Metabolic process<br>Regulation of biological process
150	XP_013963009.1	Serine/threonine-protein phosphatase 2A 55 kDa regulatory subunit B alpha isoform isoform X1	51,7	19,096	6	16	Regulation of biological process
151	XP_852423.3	Dipeptidyl peptidase 3 isoform X1	84,6	18,881	4	8	Metabolic process

152	XP_543845.5	Ubiquitin carboxyl-terminal hydrolase 5 isoform X1	98,8	18,831	7	10	Metabolic process
153	XP_535270.3	Transportin-1	106,1	18,357	5	6	Transport
154	XP_533275.2	DNA damage-binding protein 1	126,9	18,276	8	9	Cell organization and biogenesis<br>Metabolic process<br>Regulation of biological process<br>Response to stimulus
155	XP_850669.3	3'(2'),5'-bisphosphate nucleotidase 1 isoform X2	39,5	18,248	6	21	Metabolic process
156	XP_005626078.1	Eukaryotic translation initiation factor 5b isoform x1	138,6	18,176	3	6	Cell organization and biogenesis<br>Metabolic process<br>Regulation of biological process
157	XP_005634087.1	Importin-5	117	18,121	6	8	Response to stimulus<br>Transport
158	XP_022264002.1	Plastin-2 isoform X1	75,9	17,98	4	6	Cell organization and biogenesis
159	XP_013965994.2	Histone H2A type 1-E	16,7	17,798	5	18	Cell organization and biogenesis<br>Regulation of biological process
160	XP_022263484.1	Protocadherin-16	345,4	17,771	7	3	Cell differentiation<br>Cell organization and biogenesis<br>Cell proliferation<br>Regulation of biological process<br>Response to stimulus

161	XP_022269204.1	PDZ and LIM domain protein 5 isoform X1	78,8	17,668	6	11	Development<br>Cell organization and biogenesis
162	XP_536594.2	14-3-3 protein theta-like	27,8	17,576	4	11	Cell differentiation<br>Cell organization and biogenesis<br>Regulation of biological process<br>Response to stimulus
163	XP_022272210.1	Tubulin-specific chaperone D, partial	128,2	17,434	5	6	Cell organization and biogenesis
164	XP_542705.2	1-phosphatidylinositol 4,5-bisphosphate phosphodiesterase delta-1 isoform X2	88,2	17,415	7	10	Metabolic process<br>Regulation of biological process<br>Response to stimulus
165	XP_022265515.1	Glypican-1	65,4	17,314	5	8	Cell proliferation<br>Metabolic process<br>Regulation of biological process
166	XP_005622713.1	Calpain-2 catalytic subunit	76,2	17,3	6	10	Metabolic process<br>Regulation of biological process<br>Response to stimulus
167	XP_532617.2	Purine nucleoside phosphorylase	32,3	17,259	7	23	Metabolic process<br>Regulation of biological process<br>Response to stimulus<br>Transport
168	XP_022260602.1	D-3-phosphoglycerate dehydrogenase isoform X1	56,7	17,092	6	13	Metabolic process

169	XP_022272071.1	Ubiquitin carboxyl-terminal hydrolase isozyme L1	32,9	16,976	4	18	Metabolic process
170	XP_531839.2	Exportin-1 isoform X1	123,1	16,907	5	6	Regulation of biological process<br>Transport
171	XP_534044.4	Eukaryotic translation initiation factor 3 subunit F	37,2	16,897	4	14	Cell organization and biogenesis<br>Metabolic process<br>Regulation of biological process
172	XP_013973141.1	Eukaryotic peptide chain release factor subunit 1 isoform x1	51,6	16,811	5	13	Cell organization and biogenesis
173	XP_022270802.1	Insulin-like growth factor-binding protein 2 isoform X1	23,1	16,735	5	25	Metabolic process
174	XP_533130.2	Eukaryotic initiation factor 4A-III	46,8	16,653	4	9	Metabolic process<br>Regulation of biological process
175	XP_022281964.1	CD109 antigen isoform X1	162,1	16,6	5	4	Regulation of biological process
176	XP_005640223.1	Histone H2B type 2-E-like	24,5	16,529	5	19	Regulation of biological process
177	XP_543726.2	Twinfilin-1	40,1	16,395	5	16	Cell organization and biogenesis<br>Regulation of biological process
178	XP_022260274.1	Peroxidasin homolog	164,9	16,322	4	4	Metabolic process
179	XP_003639222.1	Proteasome subunit alpha type-7-like isoform X1	27,9	16,274	4	19	Metabolic process

180	XP_535824.3	26S proteasome non-ATPase regulatory subunit 2	100,2	16,213	6	7	Metabolic process<br>Regulation of biological process
181	XP_541490.4	AP-2 complex subunit alpha-1 isoform X1	107,6	15,953	6	6	Transport
182	XP_537983.2	Eukaryotic translation initiation factor 2 subunit 3	51	15,767	4	14	Cell organization and biogenesis<br>Metabolic process<br>Regulation of biological process
183	AAR97726.1	Beta amyloid precursor protein isoform APP770	86,9	15,761	5	7	Regulation of biological process
184	XP_848710.2	Poly(U)-binding-splicing factor PUF60 isoform X1	59,9	15,696	2	7	Metabolic process
185	XP_003431639.1	Isochorismatase domain-containing protein 1	32,6	15,475	3	13	Metabolic process
186	NP_001003318.1	Ras-related protein Rab-2A	23,5	15,354	5	31	Regulation of biological process<br>Response to stimulus<br>Transport
187	XP_851224.1	Cysteine-tRNA ligase, cytoplasmic isoform X1	94,6	15,263	6	8	Metabolic process
188	XP_005617874.1	Heterogeneous nuclear ribonucleoprotein R isoform X1	71,2	15,253	5	9	Metabolic process<br>Transport
189	XP_022261498.1	Neuroblast differentiation-associated protein AHNAK isoform X2	506,7	15,024	5	7	Cell differentiation

190	XP_022270322.1	Histone H2A type 1	14,1	14,938	4	21	Regulation of biological process
191	XP_022263553.1	Alpha-parvin isoform X1	41	14,892	5	18	Cell organization and biogenesis<br>Regulation of biological process
192	CAB46826.1	DNA binding protein, partial	10,8	14,599	4	45	Regulation of biological process
193	XP_543790.1	Serine-threonine kinase receptor-associated protein isoform X1	38,5	14,118	5	21	Cell organization and biogenesis<br>Regulation of biological process
194	XP_540866.2	Calpain-1 catalytic subunit	81,3	14,072	6	7	Metabolic process
195	XP_005623592.1	Eukaryotic translation initiation factor 2 subunit 1	36,1	14,068	8	23	Cell communication<br>Metabolic process<br>Regulation of biological process<br>Response to stimulus
196	XP_003638926.1	Kinesin-1 heavy chain	109,7	14,061	5	6	Cell organization and biogenesis<br>Cellular component movement<br>Metabolic process<br>Regulation of biological process<br>Transport
197	XP_022267931.1	Ubiquitin-conjugating enzyme E2 variant 2 isoform X1	18,6	14,002	5	26	Metabolic process
198	NP_001239066.1	60S acidic ribosomal protein P0	34,3	13,997	4	13	Metabolic process

199	NP_001003357.2	Hypoxanthine-guanine phosphoribosyltransferase isoform 1	24,4	13,935	3	18	Cell organization and biogenesis<br>Metabolic process<br>Regulation of biological process
200	XP_848221.2	Palladin isoform X1	129,7	13,909	5	4	Cell proliferation<br>Cell growth<br>Cell organization and biogenesis<br>Development<br>Regulation of biological process<br>Response to stimulus
201	XP_851694.4	Aldehyde dehydrogenase family 16 member A1 isoform X1	92,1	13,901	4	7	Metabolic process
202	XP_013972766.1	Golgi membrane protein 1 isoform X1	47,1	13,53	5	15	Metabolic process
203	XP_536873.3	Actin-related protein 2/3 complex subunit 1A	41,5	13,499	5	14	Cell organization and biogenesis<br>Regulation of biological process
204	NP_001013013.1	F-actin-capping protein subunit alpha-2	33	13,394	3	15	Cell organization and biogenesis<br>Regulation of biological process
205	XP_022266060.1	Bifunctional 3'-phosphoadenosine 5'-phosphosulfate synthase 2	75,1	13,392	5	10	Metabolic process
206	XP_005619548.1	Neural cell adhesion molecule 1 isoform X4	120,8	13,263	5	6	Cell organization and biogenesis<br>Metabolic process<br>Regulation of biological process<br>Response to stimulus

207	NP_001300733.1	CD166 antigen precursor	64,8	13,226	6	8	Cell growth<br>Cellular component movement<br>Response to stimulus
208	XP_003431682.1	Catenin alpha-1	100,1	13,116	4	6	Cell organization and biogenesis<br>Regulation of biological process<br>Response to stimulus
209	XP_003434941.1	Thrombospondin-3 isoform X1	104,1	13,032	4	4	Regulation of biological process
210	XP_855529.3	Cysteine-rich protein 2	22,6	12,989	2	25	Regulation of biological process
211	NP_001277045.1	Aspartate aminotransferase, cytoplasmic	46,4	12,98	6	16	Metabolic process<br>Regulation of biological process<br>Response to stimulus
212	XP_534969.3	Retinol-binding protein 4	22,9	12,861	4	21	Cell proliferation<br>Development<br>Cellular homeostasis<br>Metabolic process<br>regulacion of biological process
213	XP_852154.1	Alpha-soluble NSF attachment protein isoform X1	33,2	12,821	4	17	Cell communication<br>Cell differentiation<br>Cell organization and biogenesis<br>Regulation of biological process<br>Transport

214	XP_022279697.1	Nascent polypeptide-associated complex subunit alpha isoform X1	24,6	12,658	2	13	Development<br>Metabolic process<br>Regulation of biological process
215	XP_537159.3	Actin-related protein 2/3 complex subunit 5	16,3	12,554	2	17	Cell organization and biogenesis<br>Regulation of biological process
216	XP_848238.3	Protein disulfide-isomerase A4	72,4	12,07	3	6	Cellular homeostasis<br>Metabolic process<br>Regulation of biological process<br>Response to stimulus
217	XP_022282179.1	Laminin subunit alpha-4 isoform X1	194	11,927	7	5	Cell organization and biogenesis<br>Regulation of biological process
218	XP_005624492.1	Proteasome activator complex subunit 3 isoform X1	30,9	11,776	3	12	Regulation of biological process
219	XP_537403.4	Sec1 family domain-containing protein 1 isoform X1	72,4	11,773	3	8	Transport
220	XP_005616573.1	Sodium/potassium-Transporting ATPase subunit alpha-3 isoform X1	113	11,721	3	4	Metabolic process<br>Regulation of biological process
221	XP_537523.2	Activator of 90 kDa heat shock protein ATPase homolog 1	38,3	11,712	4	16	Regulation of biological process<br>Response to stimulus
222	XP_535738.1	Transgelin-3	22,4	11,694	3	15	Regulation of biological process
223	XP_848288.1	Phosphoserine phosphatase	25	11,66	4	20	Metabolic process

224	XP_022282006.1	Phosphoacetylglucosamine mutase isoform X1	62,4	11,66	3	6	Metabolic process
225	XP_538746.2	Sialic acid synthase	40,2	11,573	4	18	Metabolic process
226	XP_013973187.1	Septin-8 isoform X6	55,9	11,555	5	10	Regulation of biological process<br>Response to stimulus
227	XP_005622662.1	Heterogeneous nuclear ribonucleoprotein U isoform X1	79,6	11,432	4	6	Metabolic process<br>Transport
228	XP_005632596.1	Acylamino-acid-releasing enzyme isoform X1	82,6	11,392	2	5	Metabolic process
229	NP_001239095.1	Myosin regulatory light polypeptide 9	19,8	11,372	5	34	Metabolic process
230	XP_005619094.1	Protein Transport protein Sec24C isoform X1	121,2	11,276	6	7	Cell organization and biogenesis<br>Transport
231	NP_001271363.1	Protein NDRG1	41,7	11,07	3	12	Cell organization and biogenesis<br>Regulation of biological process<br>Response to stimulus
232	XP_857957.1	Heterogeneous nuclear ribonucleoprotein D0 isoform X1	38,4	11,054	5	17	Regulation of biological process
233	XP_858593.1	AP-2 complex subunit mu isoform X1	49,4	11,006	5	11	Transport

234	XP_005641798.1	Septin-6 isoform X1	49,5	11,004	4	12	Regulation of biological process<br>Response to stimulus
235	XP_536791.2	Splicing factor 3B subunit 3	135,5	10,972	4	4	Metabolic process<br>Regulation of biological process
236	XP_005639922.1	Importin subunit alpha-3 isoform X1	57,9	10,762	3	11	Response to stimulus<br>Transport
237	XP_022266143.1	Actin-related protein 2/3 complex subunit 3 isoform X1	22	10,699	3	17	Cell organization and biogenesis<br>Cellular component movement<br>Regulation of biological process
238	XP_022279753.1	Proliferation-associated protein 2G4 isoform X1	46,1	10,636	6	14	Cell growth<br>Regulation of biological process
239	XP_534612.2	COP9 signalosome complex subunit 8	23,2	10,575	4	30	Cell organization and biogenesis<br>Metabolic process
240	XP_532570.3	Vacuolar protein sorting-associated protein 35 isoform X1	91,6	10,49	5	8	Cell organization and biogenesis<br>Regulation of biological process<br>Transport
241	XP_851543.1	Protein arginine n-methyltransferase 1 isoform x1	42,4	10,463	5	18	Cell organization and biogenesis<br>Metabolic process<br>Regulation of biological process

242	XP_537306.2	Ubiquitin carboxyl-terminal hydrolase 14 isoform X1	61,4	10,308	5	12	Cell communication<br>Metabolic process<br>Regulation of biological process
243	XP_533699.3	SUMO-activating enzyme subunit 2 isoform X1	71,2	10,282	5	7	Metabolic process
244	XP_005641311.1	ATP-dependent RNA helicase DDX3X isoform X1	73,4	10,273	4	7	Cell organization and biogenesis<br>Metabolic process<br>Regulation of biological process
245	XP_866352.1	Dynactin subunit 1 isoform X1	141,8	10,215	6	5	Cell organization and biogenesis<br>Metabolic process
246	XP_534901.2	Complement C1r subcomponent	80,1	10,202	4	8	Metabolic process<br>Regulation of biological process<br>Response to stimulus
247	XP_864168.1	LIM and SH3 domain protein 1 isoform X1	29,8	10,154	5	20	Response to stimulus
248	XP_005634119.1	Tripeptidyl-peptidase 2 isoform X1	139,8	10,043	3	4	Metabolic process
249	XP_013973265.1	S-methyl-5'-thioadenosine phosphorylase isoform X1	35,1	10,031	2	8	Metabolic process
250	XP_003640041.1	ADP-ribosylation factor-like protein 3	20,4	10,026	3	28	Regulation of biological process<br>Response to stimulus
251	XP_013967513.1	Histone H2A.V isoform X3	13,5	9,954	4	31	Regulation of biological process

252	XP_533902.3	Deoxyribonuclease-2-alpha	40	9,888	2	10	Metabolic process
253	XP_547357.2	Aminopeptidase B isoform X1	72,2	9,815	4	7	Metabolic process
254	XP_005615785.1	Vasodilator-stimulated phosphoprotein isoform X1	40,5	9,581	3	10	Cell organization and biogenesis
255	XP_022264450.1	Profilin-2	9,8	9,568	3	34	Cell organization and biogenesis<br>Regulation of biological process
256	XP_005630374.1	Latent-transforming growth factor beta-binding protein 1 isoform X1	172,1	9,47	3	3	Cell death<br>Cell differentiation<br>Cell growth<br>Cell organization and biogenesis<br>Cell proliferation<br>Cellular component movement<br>Metabolic process<br>Regulation of biological process<br>Response to stimulus<br>Transport
257	XP_853634.1	Calpain small subunit 1 isoform X1	28,4	9,341	5	18	Metabolic process
258	XP_536971.1	Eukaryotic peptide chain release factor GTP-binding subunit ERF3A isoform X1	55,7	9,334	4	11	Regulation of biological process<br>Response to stimulus
259	XP_531727.4	Adenylosuccinate lyase isoform X2	55,4	9,264	3	9	Metabolic process

260	XP_535007.2	Glutathione S-transferase omega-1	27,4	9,135	4	17	Cellular homeostasis<br>Metabolic process<br>Regulation of biological process<br>Response to stimulus
261	XP_852849.3	UMP-CMP kinase isoform X1	25,8	8,961	3	14	Metabolic process
262	XP_005634815.1	Destrin	16,6	8,96	3	20	Cell organization and biogenesis<br>Regulation of biological process
263	XP_542503.3	Switch-associated protein 70 isoform X1	68,7	8,928	3	5	Cell organization and biogenesis<br>Cellular homeostasis<br>Metabolic process<br>Regulation of biological process
264	XP_533702.2	XAA-Pro dipeptidase isoform X1	54,8	8,927	5	12	Metabolic process
265	XP_537755.3	Bleomycin hydrolase	52,4	8,898	2	5	Metabolic process<br>Response to stimulus
266	XP_022269465.1	Follistatin-related protein 1	34,2	8,811	3	9	Response to stimulus<br>Transport
267	XP_013974943.1	Heterogeneous nuclear ribonucleoproteins C1/C2 isoform X1	35,1	8,748	3	7	Metabolic process<br>Transport
268	XP_854103.1	Hsp90 co-chaperone Cdc37	44,6	8,726	2	7	Metabolic process<br>Regulation of biological process

269	XP_005633450.1	Phosphatidylinositol-binding clathrin assembly protein isoform X1	77,1	8,574	4	6	Metabolic process<br>Regulation of biological process
270	NP_001238875.1	Microtubule-associated protein RP/EB family member 1	30	8,529	2	9	Regulation of biological process
271	NP_001238964.1	Mitogen-activated protein kinase 3	43	8,473	2	5	Metabolic process
272	XP_003639748.1	Translin	26,1	8,45	4	21	Metabolic process
273	XP_532275.2	Proteasome subunit beta type-1	26,4	8,368	2	10	Metabolic process
274	XP_022268163.1	Inositol monophosphatase 1 isoform X1	31,1	8,304	4	15	Metabolic process
275	XP_022263960.1	Importin subunit alpha-4	55	8,289	3	8	Transport
276	XP_013966928.1	PDZ and LIM domain protein 7-like	49,4	8,266	3	8	Development<br>Cell organization and biogenesis
277	XP_852582.2	6-phosphogluconolactonase	27,5	8,24	3	15	Metabolic process
278	Q8WMS6.1	Serine/threonine-protein phosphatase PP1-alpha catalytic subunit	37,5	8,239	3	12	Cell division<br>Metabolic process<br>Regulation of biological process<br>Response to stimulus
279	NP_001239100.1	Nucleophosmin	32,6	8,204	4	16	Regulation of biological process

280	XP_022259014.1	Ubiquitin-conjugating enzyme E2 N isoform X1	17,2	8,198	2	14	Metabolic process
281	NP_001181898.1	Heme oxygenase 1	33	8,021	3	13	Cell organization and biogenesis<br>Cellular homeostasis<br>Metabolic process<br>Regulation of biological process<br>Response to stimulus
282	XP_547771.2	Cofilin-2 isoform X1	18,7	7,977	3	20	Cell organization and biogenesis<br>Cellular homeostasis<br>Regulation of biological process
283	XP_005615306.1	Utrophin isoform X1	397,2	7,915	4	1	Transport
284	NP_001076836.1	Rho GTPase-activating protein 1	50,6	7,862	4	8	Regulation of biological process<br>Response to stimulus<br>Transport
285	XP_536085.2	Phenylalanine--tRNA ligase beta subunit isoform X1	66,2	7,701	4	6	Metabolic process
286	XP_849975.1	Probable ATP-dependent RNA helicase DDX6	54,4	7,69	3	9	Cell organization and biogenesis<br>Metabolic process<br>Regulation of biological process
287	XP_022271203.1	40S ribosomal protein S4, X isoform isoform X1	27,5	7,679	5	20	Metabolic process
288	XP_848570.2	Prolyl 3-hydroxylase 1 isoform X3	83,1	7,58	3	5	Metabolic process

289	XP_854473.1	Beta-centractin isoform X1	42,3	7,559	2	9	Cell organization and biogenesis<br>Cellular component movement<br>Regulation of biological process
290	XP_537428.1	Peptidyl-prolyl cis-trans isomerase FKBP3 isoform X1	25,2	7,513	3	9	Metabolic process
291	XP_849241.1	Proteasome subunit beta type-6	25,5	7,512	3	13	Metabolic process
292	NP_001138591.1	DNA-(apurinic or apyrimidinic site) lyase	35,4	7,5	3	13	Metabolic process<br>Response to stimulus
293	XP_854809.3	Glycogen [starch] synthase, muscle	83,5	7,491	4	5	Metabolic process
294	XP_022276296.1	Far upstream element-binding protein 1 isoform X1	75,2	7,486	3	5	Regulation of biological process
295	XP_022270477.1	Beta-hexosaminidase subunit beta	60,5	7,483	3	6	Metabolic process
296	XP_548200.3	Leucine-rich repeat-containing protein 59	34,8	7,461	2	8	Regulation of biological process<br>Transport
297	XP_005616666.1	Heterogeneous nuclear ribonucleoprotein l isoform x1	64	7,426	4	10	Metabolic process<br>Regulation of biological process
298	XP_850320.3	OBG-like ATPase 1	45,6	7,345	4	10	Metabolic process

299	NP_001124519.1	Adenylate kinase isoenzyme 1	23,4	7,326	3	17	Metabolic process<br>Regulation of biological process
300	NP_001020792.1	Cathepsin D precursor	44,3	7,299	2	5	Metabolic process
301	XP_545713.2	Transforming growth factor beta-2 isoform X1	50,5	7,296	2	5	Cell death<br>Cell differentiation<br>Cell growth<br>Cell organization and biogenesis<br>Cell proliferation<br>Cellular component movement<br>Metabolic process<br>Regulation of biological process<br>Response to stimulus<br>Transport
302	XP_022266799.1	COP9 signalosome complex subunit 7a isoform X1	31	7,274	2	9	Metabolic process<br>Regulation of biological process<br>Transport
303	NP_001239338.1	60S ribosomal protein L4	47,3	7,217	2	8	Metabolic process
304	XP_005626358.1	Heterogeneous nuclear ribonucleoprotein H isoform X1	51,2	7,189	3	7	Metabolic process<br>Transport
305	XP_022270187.1	Leukocyte elastase inhibitor	30,5	7,183	4	14	Regulation of biological process
306	XP_022283570.1	Splicing factor, proline- and glutamine-rich	75,6	7,166	4	7	Regulation of biological process

307	XP_005632735.1	Coatomer subunit epsilon isoform X1	34,5	7,15	2	11	Transport
308	XP_005636443.1	Phosphatidylinositol transfer protein beta isoform isoform X1	31,6	7,078	2	9	Transport
309	XP_536261.3	Protein NOXP20 isoform X1	60,6	6,995	2	5	Development
310	XP_013970154.1	Elongin-B	12,6	6,98	3	27	Metabolic process<br>Regulation of biological process
311	NP_001240670.1	Keratin 14	52,3	6,96	4	8	Cell differentiation<br>Cell organization and biogenesis
312	XP_856971.3	Glutathione s-transferase theta-1-like	27,6	6,929	3	16	Metabolic process
313	XP_013971057.1	Vesicle-associated membrane protein-associated protein A isoform X2	32,5	6,875	3	10	Metabolic process
314	XP_022261566.1	Nicotinamide phosphoribosyltransferase	59,1	6,85	2	6	Metabolic process
315	CAB46830.1	Ribosomal protein, partial	21,3	6,806	3	13	Metabolic process
316	XP_848697.1	Heterogeneous nuclear ribonucleoprotein F	45,6	6,795	2	6	Regulation of biological process
317	XP_536263.2	Phosphoglucomutase-2	68,4	6,738	5	10	Metabolic process
318	XP_013977447.1	Laminin subunit beta-2 isoform X1	203,7	6,728	4	2	Metabolic process

319	NP_001003082.1	Metalloproteinase inhibitor 2 precursor	24,3	6,724	2	10	Regulation of biological process<br>Response to stimulus
320	XP_533139.2	Semaphorin-3C	85,1	6,704	3	4	Cellular component movement<br>Regulation of biological process<br>Response to stimulus
321	XP_005620233.2	Myosin phosphatase Rho-interacting protein	258	6,657	4	3	Metabolic process
322	XP_862258.2	Alpha-crystallin B chain	20,1	6,646	2	13	Cell death<br>Cell organization and biogenesis<br>Regulation of biological process<br>Response to stimulus
323	NP_001238887.1	Ribosomal protein L18	21,6	6,589	2	14	Metabolic process
324	XP_533751.3	LIM and cysteine-rich domains protein 1	40,8	6,587	2	7	Regulation of biological process
325	XP_022275658.1	Laminin subunit alpha-2	347,1	6,57	3	1	Cell organization and biogenesis<br>Metabolic process<br>Regulation of biological process<br>Transport
326	NP_001162159.1	Testin	48	6,554	3	8	Regulation of biological process

327	XP_535328.3	Eukaryotic translation initiation factor 3 subunit I	36,4	6,488	4	13	Cell communication<br>Metabolic process<br>Regulation of biological process<br>Response to stimulus
328	XP_013974359.2	Peptidyl-prolyl cis-trans isomerase FKBP9	59,4	6,458	3	6	Metabolic process
329	XP_005616589.1	Platelet-activating factor acetylhydrolase IB subunit gamma isoform X1	25,8	6,449	3	15	Metabolic process
330	XP_848865.1	ATP-dependent RNA helicase DDX1 isoform X1	82,4	6,445	3	4	Development<br>Metabolic process<br>Regulation of biological process<br>Response to stimulus
331	XP_022261610.1	N-alpha-acetyltransferase 15, NatA auxiliary subunit isoform X1	95,3	6,445	3	6	Metabolic process
332	XP_013973801.1	N(G),N(G)-dimethylarginine dimethylaminohydrolase 2	29,7	6,426	2	11	Metabolic process
333	XP_022273696.1	PDZ and LIM domain protein 7	48,4	6,413	3	8	Development<br>Cell organization and biogenesis
334	XP_535229.3	Leucine--tRNA ligase, cytoplasmic isoform X1	134,1	6,319	4	4	Cell communication<br>Cell organization and biogenesis<br>Metabolic process<br>Regulation of biological process<br>Response to stimulus<br>Transport

335	NP_001238856.2	ADP-ribosylation factor 1	20,7	6,303	2	12	Regulation of biological process<br>Response to stimulus
336	NP_001003291.1	Intercellular adhesion molecule 1 precursor	58,1	6,3	3	7	Cellular component movement<br>Cellular homeostasis<br>Regulation of biological process<br>Response to stimulus
337	XP_005631766.1	Ribonuclease inhibitor	46,4	6,286	3	7	Regulation of biological process
338	XP_005618058.1	F-box only protein 6	36	6,232	2	7	Metabolic process
339	XP_003434442.1	Ubiquitin-conjugating enzyme E2 K isoform X1	22,4	6,212	3	18	Metabolic process
340	XP_540234.2	Sepiapterin reductase	28,6	6,172	3	14	Metabolic process
341	XP_849075.1	DCC-interacting protein 13-alpha isoform X1	79,6	6,15	2	5	Cell proliferation<br>Regulation of biological process<br>Response to stimulus
342	NP_001183974.1	Cytochrome c	11,6	6,147	2	18	Cell death<br>Metabolic process<br>Regulation of biological process<br>Transport
343	XP_022271449.1	Histone-binding protein RBBP7 isoform X1	52,2	6,131	2	4	Regulation of biological process<br>Response to stimulus

344	XP_005622469.1	ATP-dependent RNA helicase A isoform X1	141,2	6,013	3	3	Cell differentiation<br>Cell organization and biogenesis<br>Metabolic process<br>Regulation of biological process<br>Response to stimulus
345	XP_548099.3	Keratin, type I cytoskeletal 42	54	5,995	3	5	Cell organization and biogenesis<br>Cellular component movement<br>Metabolic process
346	XP_544656.2	Eukaryotic translation initiation factor 3 subunit J	29,1	5,946	2	8	Cell organization and biogenesis<br>Metabolic process<br>Regulation of biological process
347	XP_856598.1	Apoptosis inhibitor 5 isoform X1	59	5,919	2	5	Regulation of biological process
348	XP_864203.1	NEDD8-activating enzyme E1 catalytic subunit isoform X1	51,8	5,831	2	5	Metabolic process
349	XP_005642515.1	Ubiquitin-conjugating enzyme E2 D3	16,7	5,697	2	14	Metabolic process
350	XP_853535.1	Dynactin subunit 4 isoform X1	53,1	5,689	2	6	Cell organization and biogenesis<br>Cellular component movement<br>Regulation of biological process
351	XP_853320.1	Complement C1s subcomponent	77,5	5,521	2	4	Defense response<br>Metabolic process<br>Regulation of biological process<br>Response to stimulus

352	XP_003638817.1	Connective tissue growth factor	37,7	5,507	3	10	Cell communication<br>Cell differentiation<br>Cell proliferation<br>Cellular component movement<br>Metabolic process<br>Regulation of biological process<br>Response to stimulus
353	XP_544873.3	Carbonyl reductase [NADPH] 3	30,7	5,465	2	9	Metabolic process
354	XP_537850.1	60S ribosomal protein L35	14,6	5,465	2	18	Metabolic process
355	XP_003435545.2	Renin receptor isoform X2	39,4	5,452	2	6	Metabolic process
356	XP_005630014.1	Acid ceramidase	42,1	5,378	2	5	Metabolic process
357	XP_537183.1	Calcyclin-binding protein isoform X1	26,4	5,351	2	17	Metabolic process<br>Regulation of biological process<br>Response to stimulus
358	XP_531771.1	Sulfotransferase 1C4	35,4	5,294	2	7	Metabolic process
359	XP_537038.2	Glutathione S-transferase Mu 1 isoform X1	25,7	5,148	2	11	Metabolic process
360	XP_531754.2	tRNA-splicing ligase RtcB homolog isoform X1	55,1	5,022	3	7	Metabolic process
361	NP_001271404.1	Dickkopf-related protein 3 precursor	37,9	5,004	2	8	Development<br>Regulation of biological process

362	XP_536826.2	Cadherin-11 isoform X1	87,9	4,994	3	4	Cell organization and biogenesis
363	XP_536802.1	26S proteasome non-ATPase regulatory subunit 7	37	4,93	2	6	Metabolic process
364	XP_013971703.1	L-xylulose reductase	22,8	4,929	2	13	Metabolic process
365	XP_548937.2	Poly(rC)-binding protein 1-like	38,7	4,909	2	6	Metabolic process
366	XP_005641318.1	Peripheral plasma membrane protein CASK isoform X1	105	4,903	3	3	Metabolic process
367	XP_546742.3	Leucine-rich repeat-containing protein 47, partial	63,7	4,887	2	4	Regulation of biological process<br>Transport
368	XP_005619024.1	Hexokinase-1 isoform X1	104,2	4,873	3	3	Metabolic process
369	XP_022270692.1	Protein RCC2	53,5	4,865	2	5	Metabolic process<br>Regulation of biological process
370	XP_533261.3	Heterogeneous nuclear ribonucleoprotein U-like protein 2	85,1	4,806	3	4	Metabolic process<br>Transport
371	XP_013972162.1	Serine/arginine-rich splicing factor 1	27,7	4,8	3	12	Regulation of biological process
372	XP_005642262.2	Drebrin-like protein isoform X2	47,7	4,786	3	10	Cell organization and biogenesis<br>Transport

373	XP_003435250.1	Adapter molecule crk isoform X1	33,8	4,76	2	9	Regulation of biological process
374	XP_534355.3	Proliferating cell nuclear antigen	28,8	4,748	2	10	Cell differentiation<br>Metabolic process<br>Regulation of biological process<br>Response to stimulus
375	XP_013965708.1	Myosin light chain kinase, smooth muscle isoform X1	219,9	4,724	3	1	Metabolic process
376	XP_013970815.1	Farnesyl pyrophosphate synthase isoform X1	53,3	4,718	3	8	Metabolic process
377	NP_001239326.1	Quinone oxidoreductase	35,5	4,631	2	6	Metabolic process
378	XP_850595.1	Eukaryotic translation initiation factor 3 subunit D	63,9	4,618	2	5	Cell organization and biogenesis<br>Metabolic process<br>Regulation of biological process
379	XP_538635.3	Core histone macro-H2A.1 isoform X1	39,6	4,61	2	8	Cell organization and biogenesis<br>Regulation of biological process
380	XP_544475.2	Nuclear migration protein nudC	38,3	4,591	2	6	Metabolic process
381	XP_013963602.1	40S ribosomal protein S2-like	38	4,591	2	6	Metabolic process
382	XP_532315.1	Eukaryotic translation initiation factor 3 subunit H	39,9	4,574	3	11	Cell organization and biogenesis<br>Metabolic process<br>Regulation of biological process

383	XP_005640553.1	60 kDa heat shock protein, mitochondrial	61	4,572	2	6	Metabolic process<br>Response to stimulus
384	XP_013978140.1	Ras-related protein Rab-6A isoform X1	23,5	4,52	2	11	Regulation of biological process<br>Response to stimulus<br>Transport
385	XP_005641933.1	RNA-binding motif protein, X chromosome	42,3	4,482	2	5	Metabolic process
386	XP_853206.3	Exportin-2	110,5	4,395	3	4	Transport
387	XP_535061.2	Glutaredoxin-3 isoform x1	37,3	4,298	2	8	Cell organization and biogenesis<br>Cellular homeostasis<br>Metabolic process<br>Regulation of biological process
388	XP_013966048.1	Thioredoxin domain-containing protein 5	36,3	4,274	2	6	Cellular homeostasis<br>Metabolic process<br>Regulation of biological process<br>Response to stimulus<br>Transport
389	XP_013976791.1	Pre-mRNA-processing factor 19 isoform X1	57	4,234	2	5	Metabolic process
390	XP_848703.2	Dipeptidyl peptidase 2	59,9	4,234	2	4	Metabolic process
391	XP_852885.1	Insulin-like growth factor-binding protein 5	30,3	4,152	2	10	Regulation of biological process

392	XP_546712.3	Matrix remodeling-associated protein 8	49,9	4,128	2	4	Regulation of biological process
393	XP_856947.1	Matrix metalloproteinase-14 isoform X1	65,8	4,081	2	4	Cell differentiation<br>Cell proliferation<br>Cellular component movement<br>Metabolic process<br>Regulation of biological process
394	XP_022270828.1	Aspartyl aminopeptidase isoform X1	55,5	4,081	2	4	Metabolic process
395	XP_005637110.1	Branched-chain-amino-acid aminotransferase, cytosolic isoform X1	43	4,004	2	6	Metabolic process
396	XP_022274721.1	Protein DJ-1 isoform X1	25,2	3,957	2	7	Regulation of biological process
397	XP_543761.2	Heterogeneous nuclear ribonucleoprotein A3 isoform X1	39,6	3,942	2	10	Metabolic process<br>Transport
398	XP_005623989.1	HHIP-like protein 1	62,5	3,94	2	5	Metabolic process
399	XP_022279467.1	Protein SET isoform X1	34,9	3,921	2	8	Cell organization and biogenesis
400	XP_548424.4	Serine/threonine-protein phosphatase 2A activator isoform X1	36,8	3,791	2	10	Cell organization and biogenesis<br>Metabolic process<br>Regulation of biological process

401	XP_005617798.1	14-3-3 protein sigma	27,8	3,786	3	9	Cell differentiation<br>Cell organization and biogenesis<br>Regulation of biological process<br>Response to stimulus
402	XP_855006.2	sorting nexin-3	18,8	3,772	2	9	Cell organization and biogenesis<br>Regulation of biological process<br>Response to stimulus<br>Transport
403	NP_001032730.1	Beta-galactosidase precursor	74,9	3,712	2	4	Metabolic process
404	CAB46821.1	Ribosomal protein, partial	11,4	3,704	2	18	Metabolic process
405	XP_862938.3	LIM and senescent cell antigen-like-containing domain protein 1 isoform X2	44,4	3,689	2	5	Cell organization and biogenesis
406	XP_535093.1	COP9 signalosome complex subunit 5 isoform X1	37,5	3,677	2	4	Metabolic process<br>Regulation of biological process<br>Transport
407	NP_001301049.2	4F2 cell-surface antigen heavy chain	58,5	3,675	2	5	Metabolic process
408	XP_541337.3	NEDD8-conjugating enzyme Ubc12	20,9	3,626	2	10	Metabolic process
409	Q00004.1	Signal recognition particle subunit SRP68	70,2	3,615	2	4	Cell organization and biogenesis<br>Transport

410	XP_005631772.1	Reticulocalbin-1	37,6	3,426	2	9	Cell differentiation<br>Cell organization and biogenesis<br>Regulation of biological process<br>Response to stimulus
411	XP_532462.3	Tissue factor pathway inhibitor 2	26,2	3,418	2	7	Coagulation<br>Regulation of biological process
412	XP_003640265.1	Sorting nexin-12	18,9	3,412	2	9	Cell organization and biogenesis<br>Regulation of biological process<br>Response to stimulus<br>Transport
413	XP_013967776.1	Small nuclear ribonucleoprotein-associated protein N	24,6	3,346	2	6	Metabolic process
414	NP_001332969.1	Keratin, type I cytoskeletal 18	48,2	3,303	2	4	Cell organization and biogenesis<br>Cellular component movement<br>Metabolic process
415	XP_013966868.1	Proteasome subunit alpha type-4	31,2	3,295	2	6	Metabolic process
416	XP_022272846.1	Syntaxin-7 isoform X1	33,7	3,289	2	9	Cell organization and biogenesis<br>Regulation of biological process<br>Response to stimulus
417	XP_005641524.1	Non-POU domain-containing octamer-binding protein	54,1	3,239	2	4	Metabolic process<br>Regulation of biological process

418	XP_868610.1	Eukaryotic translation initiation factor 5	49	3,231	2	4	Metabolic process<br>Regulation of biological process
419	XP_537382.2	Proteasome activator complex subunit 2	27,3	3,08	2	6	Regulation of biological process
420	XP_005633996.1	Protein SGT1 homolog	38,3	2,949	2	6	Regulation of biological process
421	XP_538210.1	Prefoldin subunit 3 isoform X1	22,5	2,942	2	10	Cell organization and biogenesis<br>Metabolic process
422	XP_022279058.1	60S ribosomal protein L23a	14,8	2,86	2	7	Metabolic process
423	XP_539594.2	Trafficking protein particle complex subunit 3 isoform X1	20,3	2,718	2	13	Transport
424	XP_536798.2	IST1 homolog isoform X1	39,9	2,65	2	6	Cell division<br>Regulation of biological process<br>Transport
425	XP_532720.2	Synaptobrevin homolog YKT6	20,6	2,584	2	16	Transport
426	XP_532346.2	GDP-L-fucose synthase isoform X1	35,8	2,459	2	7	Metabolic process
427	XP_532129.3	Lactoylglutathione lyase	20,8	2,405	2	8	Cell differentiation<br>Regulation of biological process

428	XP_531981.2	Dynactin subunit 3 isoform X1	21,1	2,356	2	9	Cell organization and biogenesis<br>Cellular component movement<br>Regulation of biological process

List of specific proteins in common							
**Number**	**Accession**	**Description**	**MW [kDa]**	**Score**	**Peptides**	**Coverage [%]**	**Biological Process**
1	XP_867483.1	Filamin-A	280,5	769,799	97	44	Cell organization and biogenesis<br>Coagulation<br>Development<br>Metabolic process<br>Regulation of biological process<br>Transport
2	XP_013966456.1	Fibronectin isoform X4	261,5	579,581	64	38	Defense response<br>Regulation of biological process<br>Response to stimulus
3	XP_005627591.1	Collagen alpha-1(XII) chain isoform X1	339,3	572,849	103	38	Cell organization and biogenesis<br>Coagulation<br>Development<br>Metabolic process<br>Regulation of biological process<br>Transport
4	XP_013966458.1	Fibronectin isoform X7	251,7	566,714	62	38	Defense response<br>Regulation of biological process<br>Response to stimulus
5	XP_013973139.1	Tenascin isoform X1	259,9	438,657	59	28	Cell communication<br>Cell differentiation<br>Cell organization and biogenesis<br>Metabolic process<br>Regulation of biological process

							Response to stimulus
6	XP_853103.1	Alpha-actinin-1 isoform X1	105,4	437,76	52	56	Cell differentiation<br>Cell organization and biogenesis<br>Regulation of biological process
7	NP_001273952.1	Vimentin	53,6	342,292	46	66	Cell differentiation<br>Cell organization and biogenesis<br>Metabolic process<br>Regulation of biological process<br>Response to stimulus<br>Transport
8	XP_536395.3	Vinculin	127,2	330,402	50	48	Cell organization and biogenesis<br>Regulation of biological process
9	XP_005626844.1	Talin-1 isoform x1	271,2	322,866	65	30	Cell organization and biogenesis
10	XP_013963109.1	Collagen alpha-3(VI) chain isoform X1	344,1	273,073	72	28	Cell organization and biogenesis<br>Regulation of biological process<br>Response to stimulus
11	XP_013964909.2	Pyruvate kinase PKM isoform X1	64,3	270,247	30	51	Metabolic process
12	NP_001003187.1	collagen alpha-2(I) chain precursor	129,3	264,117	43	53	Cell organization and biogenesis<br>Regulation of biological process<br>Response to stimulus

13	XP_536735.3	alpha-enolase	49	244,552	27	45	Metabolic process<br>Regulation of biological process<br>Response to stimulus
14	XP_005621076.1	Actin, cytoplasmic 1 isoform X1	44,8	236,41	23	53	Cell organization and biogenesis
15	NP_001003090.1	Collagen alpha-1(I) chain precursor	138,7	224,982	37	39	Cell organization and biogenesis<br>Coagulation<br>Development<br>Metabolic process<br>Regulation of biological process<br>Transport
16	NP_001104237.1	Myosin-9	226,3	210,897	44	26	Cell differentiation<br>Cell organization and biogenesis<br>Cellular component movement<br>Metabolic process<br>Regulation of biological process<br>Transport
17	XP_022270652.1	Basement membrane-specific heparan sulfate proteoglycan core protein, partial	484	209,148	40	12	Cell differentiation<br>Cell organization and biogenesis<br>Defense response<br>Metabolic process<br>Regulation of biological process
18	XP_535424.1	Actin, alpha cardiac muscle 1	42	206,408	23	57	Cell organization and biogenesis<br>Cellular component movement<br>Regulation of biological process

19	XP_013963753.1	Dihydropyrimidinase-related protein 3 isoform X1	62,2	200,635	22	49	Cell organization and biogenesis<br>Cellular component movement<br>Regulation of biological process
20	XP_534781.2	Actin, aortic smooth muscle	42	196,197	22	57	Regulation of biological process
21	XP_005638698.1	Chondroitin sulfate proteoglycan 4	247,9	191,648	42	23	Cell proliferation<br>Cellular component movement<br>Regulation of biological process<br>Response to stimulus
22	XP_849434.1	Fructose-bisphosphate aldolase A	39,5	190,113	22	62	Metabolic process
23	XP_005619707.1	Heat shock cognate 71 kDa protein	70,9	183,99	28	40	Cell organization and biogenesis
24	CAZ17012.1	Serpin peptidase inhibitor, clade H (heat shock protein 47), member 1, (collagen binding protein 1)	46,6	171,202	20	47	Regulation of biological process
25	XP_536561.2	Transgelin	22,6	162,648	17	72	Cell differentiation<br>Development
26	NP_001124309.1	WD repeat-containing protein 1	66,3	161,838	21	46	Cell differentiation<br>Cell organization and biogenesis<br>Cellular component movement<br>Metabolic process<br>Regulation of biological process

27	XP_863385.2	78 kDa glucose-regulated protein	72,2	159,905	31	48	Cell organization and biogenesis
28	XP_860038.2	Phosphoglycerate mutase 1	28,8	146,431	11	39	Metabolic process<br>Regulation of biological process
29	XP_544610.3	Thrombospondin-1	129,5	142,486	32	24	Cell organization and biogenesis<br>Cellular component movement<br>Defense response<br>Metabolic process<br>Regulation of biological process<br>Response to stimulus
30	XP_005641475.1	Moesin isoform X1	69,7	137,124	29	43	Cell differentiation<br>Cell organization and biogenesis<br>Cell proliferation<br>Cellular component movement<br>Regulation of biological process<br>Response to stimulus
31	XP_536199.2	Ras GTPase-activating-like protein IQGAP1	189	135,654	34	25	Cell growth<br>Regulation of biological process<br>Response to stimulus
32	XP_538147.2	Plastin-3	70,7	135,494	20	35	Cell organization and biogenesis
33	XP_849992.1	Phosphoglycerate kinase 1	44,4	134,116	16	41	Metabolic process
34	ADE58430.1	Thrombospondin 2	129,3	126,724	20	18	Regulation of biological process

35	XP_535453.3	Protein disulfide-isomerase A3	56,7	126,431	25	42	Cellular homeostasis<br>Metabolic process<br>Regulation of biological process<br>Response to stimulus
36	XP_005640450.1	Collagen alpha-2(V) chain isoform X1	144,7	126,279	29	33	Cell organization and biogenesis<br>Regulation of biological process<br>Response to stimulus
37	XP_022279791.1	Prolow-density lipoprotein receptor-related protein 1 isoform X1	506,2	119,645	29	8	Metabolic process<br>Response to stimulus
38	XP_005624805.1	Clathrin heavy chain 1 isoform X1	192,3	112,222	26	19	Transport
39	XP_534084.4	L-lactate dehydrogenase A chain isoform X1	39,8	106,923	15	37	Metabolic process
40	XP_005625117.1	Tubulin beta-4B chain isoform X2	49,8	104,235	18	47	Cell organization and biogenesis
41	XP_533949.3	Elongation factor 2	95,3	101,298	21	24	Metabolic process
42	XP_849125.1	Dihydropyrimidinase-related protein 2 isoform X1	73,5	98,992	16	33	Cell organization and biogenesis<br>Cellular component movement<br>Regulation of biological process
43	XP_003639259.1	Creatine kinase B-type	42,7	98,916	13	40	Cellular homeostasis<br>Metabolic process

44	XP_532060.4	Tubulin beta chain	49,6	98,279	18	47	Cell organization and biogenesis
45	XP_853858.1	Glucose-6-phosphate isomerase	62,8	98,269	16	32	Metabolic process<br>Regulation of biological process
46	NP_001003142.2	Glyceraldehyde-3-phosphate dehydrogenase	35,8	91,697	13	44	Cell death<br>Cell organization and biogenesis<br>Metabolic process<br>Regulation of biological process
47	XP_003640008.1	Tubulin alpha-1C chain	49,9	90,226	14	33	Cell organization and biogenesis
48	NP_001273918.1	Ezrin	69,4	87,656	21	32	Cell organization and biogenesis<br>Metabolic process<br>Regulation of biological process<br>Response to stimulus<br>Transport
49	XP_022272632.1	Lactadherin	35,8	87,285	19	51	Regulation of biological process
50	XP_532154.4	Heat shock protein HSP 90-beta	83,2	85,97	26	31	Cell organization and biogenesis<br>Metabolic process<br>Regulation of biological process<br>Response to stimulus
51	XP_005618201.1	EGF-like repeat and discoidin I-like domain-containing protein 3 isoform X1	53,7	85,439	18	32	Regulation of biological process

52	NP_001273874.1	Fructose-bisphosphate aldolase C	39,3	84,592	14	47	Metabolic process
53	XP_536077.2	Tubulin alpha-4A chain isoform X1	49,9	83,07	13	29	Cell organization and biogenesis
54	XP_003434049.2	Collagen alpha-1(VI) chain	108,5	82,318	17	20	Cell differentiation<br>Cell organization and biogenesis<br>Response to stimulus
55	XP_537556.2	Cytoplasmic dynein 1 heavy chain 1	532,1	80,622	35	9	Cell organization and biogenesis<br>Cellular component movement<br>Transport
56	NP_001274091.1	Glycogen phosphorylase, liver form	97,4	78,932	23	26	Metabolic process
57	XP_852741.2	Transgelin-2	22,4	76,716	13	67	Cell differentiation
58	XP_537659.4	Puromycin-sensitive aminopeptidase isoform X1	96,9	75,846	20	26	Metabolic process<br>Response to stimulus
59	XP_005623992.2	Heat shock protein HSP 90-alpha	71,5	75,423	22	28	Metabolic process<br>Response to stimulus
60	NP_001183983.1	Triosephosphate isomerase	26,7	73,223	11	45	Metabolic process
61	NP_001184045.1	Elongation factor 1-alpha 1	50,1	70,419	11	27	Metabolic process

62	XP_545884.4	Cell migration-inducing and hyaluronan-binding protein isoform X1	152,8	69,829	22	21	Cell organization and biogenesis
63	XP_534893.3	Alpha-2-macroglobulin	165,1	66,783	21	16	Cell differentiation<br>Regulation of biological process
64	XP_013966859.1	Ubiquitin-like modifier-activating enzyme 1	119,3	65,216	15	16	Metabolic process
65	XP_535868.3	Tubulin beta-2A chain	41,9	65,066	13	37	Cell organization and biogenesis
66	NP_001184024.1	Plasminogen activator inhibitor 1 precursor	45,3	64,881	16	50	Defense response<br>Regulation of biological process<br>Response to stimulus
67	NP_001238871.1	Eukaryotic initiation factor 4A-I	46,1	63,504	14	33	Metabolic process<br>Regulation of biological process
68	XP_848565.1	Adenylyl cyclase-associated protein 1 isoform X1	51,4	60,88	11	21	Cell organization and biogenesis<br>Regulation of biological process<br>Response to stimulus
69	NP_001239094.1	Peroxiredoxin-1	22,1	60,267	16	50	Cellular homeostasis<br>Metabolic process<br>Regulation of biological process
70	NP_001002961.1	Annexin A2	38,6	59,838	16	45	Regulation of biological process

71	AAF78600.1	Cyclophilin A, partial	16,9	59,21	9	51	Metabolic process
72	NP_001003184.1	Rab GDP dissociation inhibitor beta	50,3	58,519	13	29	Metabolic process<br>Regulation of biological process<br>Response to stimulus<br>Transport
73	NP_001003067.2	Heat shock 70 kDa protein 1	70	58,158	16	25	Cell organization and biogenesis<br>Metabolic process<br>Regulation of biological process
74	XP_852754.1	14-3-3 protein beta/alpha	27,8	57,94	9	28	Regulation of biological process<br>Transport
75	XP_005637869.2	Serine protease HTRA1	37,6	57,11	10	34	Metabolic process
76	XP_853577.1	Elongation factor 1-gamma	50,1	57,099	10	23	Metabolic process
77	XP_537245.3	T-complex protein 1 subunit gamma isoform X1	60,6	55,786	15	33	Metabolic process<br>Regulation of biological process<br>Transport
78	XP_022268859.1	Collagen alpha-2(VI) chain isoform X1	93,5	53,83	13	15	Cell organization and biogenesis
79	XP_860433.2	Tubulin beta-6 chain	49,8	52,007	13	33	Cell organization and biogenesis

80	XP_853454.1	Annexin A6 isoform X1	75,8	51,399	18	32	Metabolic process<br>Regulation of biological process<br>Response to stimulus
81	XP_541181.1	T-complex protein 1 subunit alpha isoform X1	60,2	49,965	13	25	Metabolic process<br>Regulation of biological process<br>Transport
82	XP_853309.2	Actin-related protein 3	42	49,916	12	32	Cell organization and biogenesis<br>Regulation of biological process
83	NP_001239093.1	L-lactate dehydrogenase B chain	36,6	47,729	11	33	Metabolic process
84	XP_535411.2	6-phosphogluconate dehydrogenase, decarboxylating	53,1	46,68	12	29	Metabolic process
85	XP_548489.4	Inter-alpha-trypsin inhibitor heavy chain H3	105,3	46,279	7	8	Metabolic process
86	NP_001003327.1	Endoplasmin precursor	92,5	46,181	13	16	Metabolic process<br>Response to stimulus
87	XP_005625672.1	T-complex protein 1 subunit beta isoform X1	61,6	46,017	14	29	Metabolic process<br>Regulation of biological process<br>Transport
88	XP_538929.2	Inactive tyrosine-protein kinase 7	118,3	45,484	11	13	Metabolic process<br>Regulation of biological process<br>Response to stimulus

89	NP_001003370.1	Clusterin precursor	51,8	45,428	12	24	Metabolic process<br>Regulation of biological process<br>Response to stimulus
90	XP_544493.2	Chloride intracellular channel protein 4	28,7	43,462	7	31	Metabolic process<br>Regulation of biological process<br>Transport
91	NP_001003392.1	Keratin, type II cytoskeletal 1	63,8	43,426	11	15	Cell organization and biogenesis<br>Cellular component movement<br>Metabolic process
92	XP_537190.4	Peroxiredoxin-6	17,6	42,707	10	68	Cellular homeostasis<br>Metabolic process<br>Regulation of biological process
93	XP_022262838.1	Complement C3	176	42,146	12	5	Metabolic process<br>Regulation of biological process<br>Response to stimulus
94	XP_003433179.1	Glycogen phosphorylase, brain form	96,6	41,826	11	13	Metabolic process
95	XP_865342.1	Phosphoglucomutase-1 isoform X2	61,4	41,392	15	27	Metabolic process
96	XP_546998.3	Fascin	54,6	40,905	10	23	Cell organization and biogenesis<br>Cellular component movement<br>Regulation of biological process

97	XP_533520.2	Phosphoserine aminotransferase isoform X2	40,4	40,401	9	24	Metabolic process<br>Regulation of biological process<br>Response to stimulus
98	XP_022280742.1	Gelsolin	88	40,271	12	14	Cell organization and biogenesis<br>Metabolic process<br>Regulation of biological process
99	XP_534490.2	Periostin isoform X1	93	39,708	14	20	Cell communication<br>Cell organization and biogenesis<br>Regulation of biological process<br>Response to stimulus
100	XP_548162.2	Importin subunit beta-1	97,2	39,207	12	16	Cell organization and biogenesis<br>Cellular component movement<br>Regulation of biological process<br>Response to stimulus<br>Transport
101	XP_003433473.2	14-3-3 protein eta	28,2	37,881	8	24	Metabolic process<br>Regulation of biological process<br>Response to stimulus<br>Transport
102	XP_534388.2	Adenosylhomocysteinase	47,7	34,342	11	20	Metabolic process<br>Transport
103	XP_013972035.1	ATP-citrate synthase isoform X4	126	33,774	12	15	Metabolic process

104	NP_001003185.1	Rab GDP dissociation inhibitor alpha	50,5	33,26	10	20	Metabolic process<br>Regulation of biological process<br>Response to stimulus<br>Transport
105	XP_013970599.1	Laminin subunit gamma-1	164,5	33,125	10	9	Cell organization and biogenesis<br>Cellular component movement<br>Regulation of biological process
106	NP_001041557.1	Phosphatidylethanolamine-binding protein 1	20,9	32,519	7	55	Regulation of biological process
107	XP_545634.2	Bifunctional purine biosynthesis protein purH	64,7	32,457	12	26	Metabolic process
108	XP_013972063.1	Keratin, type I cytoskeletal 10 isoform X1	62,2	32,416	13	17	Cell organization and biogenesis<br>Cellular component movement<br>Metabolic process
109	XP_540905.2	Neutral alpha-glucosidase AB isoform X1	108,8	32,204	10	14	Metabolic process
110	XP_013970643.1	T-complex protein 1 subunit zeta-like	57,9	31,584	11	20	Metabolic process<br>Regulation of biological process<br>Transport
111	XP_853454.1	Annexin A6 isoform X1	75,8	31,043	11	21	Metabolic process<br>Regulation of biological process<br>Response to stimulus

112	XP_849933.1	Rho GDP-dissociation inhibitor 1	23,4	30,708	7	34	Regulation of biological process<br>Response to stimulus
113	XP_536509.3	Threonine-tRNA ligase, cytoplasmic	83,6	29,961	12	19	Metabolic process
114	XP_533066.2	F-actin-capping protein subunit alpha-1	33	29,916	6	35	Cell organization and biogenesis<br>Regulation of biological process
115	XP_539716.1	Lumican	38,3	29,579	8	21	Cell organization and biogenesis<br>Regulation of biological process
116	XP_005616279.1	serine/threonine-protein phosphatase 2A 65 kDa regulatory subunit A alpha isoform isoform X1	65,4	29,281	9	19	Cell organization and biogenesis
117	XP_852889.1	T-complex protein 1 subunit eta	59,4	28,766	7	15	Metabolic process
118	XP_536254.2	UDP-glucose 6-dehydrogenase	55	28,387	6	15	Metabolic process
119	NP_001301047.1	Annexin A5	35,9	28,05	9	29	Regulation of biological process<br>Response to stimulus
120	XP_537554.1	Tryptophan-tRNA ligase, cytoplasmic	53,8	27,738	7	20	Metabolic process<br>Regulation of biological process
121	XP_022281488.1	Complement C4-A	192,8	27,585	6	5	Regulation of biological process<br>Response to stimulus

122	NP_001188417.1	Galectin-1	14,7	27,402	7	47	Cell differentiation<br>Defense response<br>Metabolic process<br>Response to stimulus
123	XP_860499.2	Eukaryotic initiation factor 4A-II	46,4	27,311	7	18	Metabolic process<br>Regulation of biological process
124	XP_022269344.1	Septin-11 isoform X1	49,6	26,334	8	21	Regulation of biological process<br>Response to stimulus
125	XP_537476.2	C-1-tetrahydrofolate synthase, cytoplasmic isoform X2	101,2	26,009	7	10	Metabolic process
126	XP_533644.1	Apolipoprotein E	37,2	25,942	11	31	Cell organization and biogenesis<br>Cellular homeostasis<br>Metabolic process<br>Regulation of biological process<br>Response to stimulus<br>Transport
127	XP_535195.2	Inter-alpha-trypsin inhibitor heavy chain H2	106,9	25,439	5	5	Metabolic process
128	NP_001239067.1	Chloride intracellular channel protein 1	27	24,991	5	29	Regulation of biological process<br>Transport
129	XP_022278158.1	Fermitin family homolog 2	74,9	24,174	9	15	Cell organization and biogenesis<br>Metabolic process<br>Regulation of biological process<br>Transport

130	XP_545103.1	V-type proton ATPase catalytic subunit A	68,4	24,055	6	14	Cellular homeostasis<br>Metabolic process<br>Response to stimulus<br>Transport
131	XP_005625811.1	Fibulin-1 isoform X1	77,9	23,789	7	10	Cell organization and biogenesis<br>Metabolic process<br>Regulation of biological process<br>Response to stimulus
132	XP_544922.3	ATP-dependent 6-phosphofructokinase, liver type isoform X1	85,2	23,458	7	13	Cell organization and biogenesis<br>Metabolic process<br>Regulation of biological process<br>Response to stimulus
133	XP_537412.2	Proteasome subunit alpha type-6 isoform X1	27,4	22,892	7	30	Metabolic process<br>Regulation of biological process
134	XP_022279895.1	Cullin-associated nedd8-dissociated protein 1	133,5	22,884	7	7	Cell differentiation<br>Cell organization and biogenesis<br>Regulation of biological process<br>Metabolic process
135	XP_005618229.1	Thrombospondin-4	104,1	22,828	4	5	Regulation of biological process
136	XP_005626496.1	Protein-lysine 6-oxidase isoform X1	46,1	22,804	6	16	Cell organization and biogenesis<br>Metabolic process

137	XP_003639378.1	T-complex protein 1 subunit delta	58	22,585	9	20	Metabolic process<br>Regulation of biological process<br>Transport
138	XP_022278108.1	Mimecan isoform X1	42,7	22,004	9	13	Cell growth<br>Response to stimulus
139	XP_005626208.1	UTP-glucose-1-phosphate uridylyltransferase isoform x1	56,9	21,989	10	24	Metabolic process
140	XP_537187.4	Antithrombin-III	52,4	21,887	7	12	Regulation of biological process
141	NP_001239345.1	Aldose reductase	35,7	21,871	6	25	Metabolic process
142	NP_001003026.1	Serum albumin precursor	68,6	21,801	4	5	Cell communication<br>Regulation of biological process<br>Response to stimulus<br>Transport
143	XP_848666.1	Aspartate-tRNA ligase, cytoplasmic isoform X1	57	21,793	11	23	Metabolic process
144	NP_001019808.1	Nucleoside diphosphate kinase A	17,2	21,659	4	39	Cell differentiation<br>Metabolic process<br>Regulation of biological process<br>Transport

145	XP_022261847.1	Integrin beta-1 isoform X2	88,1	21,363	8	12	Cell differentiation<br>Cell growth<br>Cell organization and biogenesis<br>Cellular component movement<br>Metabolic process<br>Regulation of biological process<br>Response to stimulus<br>Transport
146	XP_867310.2	Calreticulin	48,2	20,81	6	18	Metabolic process<br>Regulation of biological process
147	XP_546076.2	Nidogen-1	136,3	20,806	7	7	Cell organization and biogenesis<br>Regulation of biological process
148	NP_001003063.1	Serine/threonine-protein phosphatase 2A catalytic subunit alpha isoform	35,6	20,411	4	19	Metabolic process
149	XP_005619977.3	Profilin-1	12,7	20,349	3	33	Cell organization and biogenesis<br>Regulation of biological process
150	XP_537840.2	Angiopoietin-related protein 2	57,1	20,225	6	15	Metabolic process<br>Regulation of biological process<br>Response to stimulus
151	NP_001002987.1	Secreted frizzled-related protein 2 precursor	33,3	20,144	4	11	Cell communication<br>Cell differentiation<br>Development<br>Regulation of biological process<br>Response to stimulus

152	XP_005617240.2	ATP-dependent 6-phosphofructokinase, platelet type isoform X1	88	18,892	6	10	Metabolic process
153	XP_013962772.1	Proteasome subunit alpha type-7	20,2	18,437	4	28	Metabolic process
154	XP_859401.1	Procollagen C-endopeptidase enhancer 1	48,6	17,961	5	16	Metabolic process<br>Regulation of biological process
155	XP_003640345.1	Histone H2B type 1-like, partial	25,5	17,869	5	17	Cell organization and biogenesis<br>Regulation of biological process
156	XP_537577.2	cAMP-dependent protein kinase type I-alpha regulatory subunit	43	17,844	4	14	Regulation of biological process
157	XP_532408.2	Afamin	69,1	17,752	4	8	Regulation of biological process<br>Response to stimulus<br>Transport
158	XP_536047.2	Isocitrate dehydrogenase [NADP] cytoplasmic	46,8	17,725	5	14	Metabolic process<br>Regulation of biological process<br>Response to stimulus
159	NP_001003316.1	Ras-related protein Rab-7a	23,5	17,495	5	28	Cell organization and biogenesis<br>Cellular homeostasis<br>Metabolic process<br>Regulation of biological process<br>Response to stimulus<br>Transport

160	XP_022275295.1	14-3-3 protein gamma	26,5	17,064	5	20	Cell differentiation<br>Cell organization and biogenesis<br>Regulation of biological process<br>Response to stimulus
161	XP_022262967.1	Thimet oligopeptidase isoform X1	78,4	16,909	5	10	Metabolic process
162	CAB46814.1	Ubiquitin-ribosomal protein L40 fusion protein	14,7	16,181	5	41	Metabolic process
163	P02648.2	Apolipoprotein A	30,2	16,086	2	6	Cell organization and biogenesis<br>Metabolic process<br>Regulation of biological process<br>Response to stimulus<br>Transport
164	XP_854399.2	Ras-related protein Rab-11B	24,5	15,951	8	36	Metabolic process<br>Regulation of biological process<br>Response to stimulus<br>Transport
165	NP_001003306.1	Sodium/potassium-Transporting ATPase subunit alpha-1 precursor	112,6	15,849	5	6	Regulation of biological process<br>Transport
166	NP_001192025.1	Ubiquitin-conjugating enzyme E2 variant 1	16,5	15,683	5	30	Metabolic process
167	NP_001239347.1	Hsc70-interacting protein	41,4	15,675	4	12	Regulation of biological process<br>Response to stimulus

168	XP_013966259.1	Integrin alpha-V	111,1	15,525	6	8	Cell differentiation<br>Cell organization and biogenesis<br>Cellular component movement<br>Regulation of biological process<br>Response to stimulus
169	XP_849047.1	ADP-ribosylation factor 4	20,5	15,353	4	27	Cellular component movement<br>Metabolic process<br>Regulation of biological process<br>Response to stimulus<br>Transport
170	XP_537215.1	Fumarate hydratase, mitochondrial	54,4	15,147	3	10	Metabolic process
171	XP_851250.1	Ras-related protein Rap-1b	20,8	14,913	4	20	Cell proliferation<br>Regulation of biological process<br>Response to stimulus
172	XP_541532.2	EH domain-containing protein 2	61,2	14,895	7	12	Cell organization and biogenesis<br>Metabolic process<br>Regulation of biological process<br>Transport
173	XP_022270931.1	Neuropilin-2 isoform X1	104,6	14,647	8	10	Cell differentiation<br>Cell growth<br>Cell organization and biogenesis<br>Cellular component movement<br>Development<br>Regulation of biological process<br>Response to stimulus<br>Transport

174	NP_001273899.1	Annexin A1	38,6	14,389	4	17	Cell differentiation<br>Cell organization and biogenesis<br>Cellular component movement<br>Defense response<br>Metabolic process<br>Regulation of biological process<br>Response to stimulus<br>Transport
175	XP_003432617.1	Echinoderm microtubule-associated protein-like 2 isoform X1	91,6	13,876	5	8	Regulation of biological process
176	XP_013973355.1	Collagen alpha-1(XV) chain isoform X1	150,2	13,554	4	3	Cell organization and biogenesis<br>Coagulation<br>Development<br>Metabolic process<br>Regulation of biological process<br>Transport
177	NP_001239084.1	Alcohol dehydrogenase class-3	39,6	12,933	2	8	Metabolic process
178	XP_013976235.2	Laminin subunit beta-1	181,8	12,09	5	3	Cell differentiation<br>Cell organization and biogenesis<br>Cellular component movement<br>Metabolic process<br>Regulation of biological process<br>Transport
179	XP_005625812.1	Fibulin-1 isoform X2	75,2	11,902	3	4	Cell organization and biogenesis<br>Metabolic process<br>Regulation of biological process<br>Response to stimulus

180	XP_863089.1	Vitamin D-binding protein	52,9	11,697	2	5	Transport
181	NP_001239097.1	Alcohol dehydrogenase [NADP(+)]	36,6	11,679	6	18	Metabolic process
182	XP_005619064.1	Prolyl 4-hydroxylase subunit alpha-1 isoform X2	61	11,567	3	7	Metabolic process
183	XP_537759.2	Glyoxalase domain-containing protein 4 isoform X1	33,5	11,064	4	17	Metabolic process
184	XP_854482.1	Platelet-activating factor acetylhydrolase IB subunit alpha	46,6	10,786	5	14	Cell communication<br>Cell differentiation<br>Cell division<br>Cell organization and biogenesis<br>Cell proliferation<br>Cellular component movement<br>Development<br>Metabolic process<br>Regulation of biological process<br>Response to stimulus<br>Transport
185	XP_546339.2	Complement component C7 isoform X1	94,9	10,766	3	5	Response to stimulus
186	NP_001332964.1	Keratin, type II cytoskeletal 5	62,8	10,757	3	5	Metabolic process
187	XP_532217.4	NADP-dependent malic enzyme	61	10,596	5	10	Metabolic process

188	XP_005621078.1	Ras-related C3 botulinum toxin substrate 1 isoform X1	23,5	10,594	4	17	Cell organization and biogenesis<br>Cellular component movement<br>Regulation of biological process<br>Response to stimulus
189	XP_536148.4	4-trimethylaminobutyraldehyde dehydrogenase	54	10,085	5	10	Metabolic process
190	XP_543444.2	Coronin-1C	53,2	10,073	3	6	Cell organization and biogenesis
191	NP_001003027.1	Alpha-fetoprotein precursor	68,7	9,821	5	7	Transport
192	XP_535835.2	Fetuin-B	42,3	9,391	3	3	Regulation of biological process
193	NP_001003364.1	Guanine nucleotide-binding protein G(i) subunit alpha-2	40,5	9,303	3	11	Cell division<br>Cell proliferation<br>Regulation of biological process<br>Response to stimulus
194	XP_540154.2	Aldose 1-epimerase isoform X1	37,5	9,189	2	8	Metabolic process
195	XP_013976696.1	Coronin-1B	54,2	9,016	3	8	Cell organization and biogenesis
196	XP_005636307.1	Tyrosine-protein phosphatase non-receptor type 11 isoform X1	68,4	8,704	5	9	Metabolic process

197	NP_001274058.1	Thy-1 membrane glycoprotein precursor	18	8,537	3	23	Cell communication<br>Cell organization and biogenesis<br>Regulation of biological process<br>Response to stimulus
198	XP_022270107.1	GTP-binding nuclear protein Ran-like	21	8,481	2	13	Regulation of biological process<br>Response to stimulus
199	XP_005633752.2	Proteasome subunit alpha type-1	29,5	8,137	4	13	Metabolic process
200	XP_013965975.2	Tubulin beta-4B chain-like	8,6	7,99	2	35	Cell organization and biogenesis
201	XP_542374.3	Hemoglobin subunit epsilon	16,1	7,571	2	13	Transport
202	NP_001273996.1	S-adenosylmethionine synthase	43,6	7,349	2	7	Cell organization and biogenesis<br>Metabolic process
203	AAM88380.1	Protein phosphatase type 1 catalytic subunit delta isoform	37,2	7,307	2	6	Cell division<br>Metabolic process<br>Regulation of biological process
204	XP_022261337.1	Ras-related protein Rab-1B isoform X1	23,2	6,617	3	16	Cell differentiation<br>Regulation of biological process<br>Response to stimulus
205	XP_537460.2	Proteasome subunit alpha type-3	28,4	6,448	2	9	Metabolic process<br>Regulation of biological process

206	XP_543263.2	V-type proton ATPase subunit B, brain isoform	56,5	6,447	3	7	Metabolic process<br>Transport
207	XP_005634088.1	FERM, RhoGEF and pleckstrin domain-containing protein 1 isoform X1	117,7	6,385	3	3	Regulation of biological process
208	XP_022276544.1	Cofilin-1-like	21,3	6,276	2	10	Cell organization and biogenesis
209	XP_022265506.1	Bone morphogenetic protein 1	113,9	6,041	3	3	Cell differentiation<br>Development<br>Metabolic process<br>Regulation of biological process
210	XP_005638220.1	Growth/differentiation factor 6	45,1	5,721	3	9	Cell differentiation<br>Cell growth
211	XP_848457.1	26S proteasome non-ATPase regulatory subunit 13	42,9	5,121	2	5	Cell organization and biogenesis<br>Metabolic process
212	XP_533057.3	Proteasome subunit beta type-4	29,1	4,873	2	7	Metabolic process<br>Regulation of biological process
213	XP_005627062.1	26S proteasome non-ATPase regulatory subunit 5, partial	55,8	4,548	3	6	Cell organization and biogenesis
214	XP_003433222.1	Pentraxin-related protein PTX3	37,5	4,417	2	6	Defense response<br>Metabolic process<br>Regulation of biological process

215	XP_866308.3	Four and a half LIM domains protein 1 isoform X3	33,7	4,417	2	6	Regulation of biological process
216	XP_005639865.1	Alpha-2-HS-glycoprotein	39,2	4,413	2	9	Defense response<br>Regulation of biological process<br>Response to stimulus
217	XP_022260873.1	Biliverdin reductase A isoform X1	39,9	3,775	2	7	Metabolic process<br>Regulation of biological process<br>Transport
218	NP_001257814.1	Hemoglobin subunit alpha-like	15,3	2,849	2	6	Transport
