# Supplementary material for: Characterization of the secretory profile and exosomes of limbal stem cells in the canine species
Source: PLoS One. 2020 Dec 29;15(12):e0244327. doi: 10.1371/journal.pone.0244327 (PMC7771867; doi:10.1371/journal.pone.0244327)
Supplement: S2 Table — (PDF) [file pone.0244327.s002.pdf]

List of specific proteins in cLSC exosomes and canine bone marrow MSCs exosomes							
**Number**	**Accession**	**Description**	**MW [kDa]**	**Score**	**Peptides**	**Coverage [%]**	**Biological Process**
1	XP_013966456.1	Fibronectin isoform X4	261,5	579,581	64	38	Defense response<br>Regulation of biological process<br>Response to stimulus
2	XP_005627591.1	Collagen alpha-1(XII) chain isoform X1	339,3	572,849	103	38	Cell organization and biogenesis<br>Coagulation<br>Development<br>Metabolic process<br>Regulation of biological process<br>Transport
3	XP_013966458.1	Fibronectin isoform X7	251,7	566,714	62	38	Defense response<br>Regulation of biological process<br>Response to stimulus
4	XP_013964909.2	Pyruvate kinase PKM isoform X1	64,3	270,247	30	51	Metabolic process
5	XP_005621076.1	Actin, cytoplasmic 1 isoform X1	44,8	236,41	23	53	Cell organization and biogénesis

6	NP_001104237.1	Myosin-9	226,3	210,897	44	26	Cell differentiation<br>Cell organization and biogenesis<br>Cellular component movement<br>Metabolic process<br>Regulation of biological process<br>Transport
7	XP_849992.1	Phosphoglycerate kinase 1	44,4	134,116	16	41	Metabolic process
8	XP_005624805.1	Clathrin heavy chain 1 isoform X1	192,3	112,222	26	19	Transport
9	XP_534084.4	L-lactate dehydrogenase A chain isoform X1	39,8	106,923	15	37	Metabolic process
10	XP_532060.4	Tubulin beta chain	49,6	98,279	18	47	Cell organization and biogénesis
11	NP_001003142.2	Glyceraldehyde-3-phosphate dehydrogenase	35,8	91,697	13	44	Cell death<br>Cell organization and biogenesis<br>Metabolic process<br>Regulation of biological process
12	XP_003640008.1	Tubulin alpha-1C chain	49,9	90,226	14	33	Cell organization and biogenesis
13	NP_001184045.1	Elongation factor 1-alpha 1	50,1	70,419	11	27	Metabolic process
14	XP_534893.3	Alpha-2-macroglobulin	165,1	66,783	21	16	Cell differentiation<br>Regulation of biological process

15	NP_001002961.1	Annexin A2	38,6	59,838	16	45	Regulation of biological process
16	NP_001003184.1	Rab GDP dissociation inhibitor beta	50,3	58,519	13	29	Metabolic process<br>Regulation of biological process<br>Response to stimulus<br>Transport
17	NP_001003067.2	Heat shock 70 kDa protein 1	70	58,158	16	25	Cell organization and biogenesis<br>Metabolic process<br>Regulation of biological process
18	XP_538929.2	Inactive tyrosine-protein kinase 7	118,3	45,484	11	13	Metabolic process<br>Regulation of biological process<br>Response to stimulus
19	XP_544493.2	Chloride intracellular channel protein 4	28,7	43,462	7	31	Metabolic process<br>Regulation of biological process<br>Transport
20	NP_001003392.1	Keratin, type II cytoskeletal 1	63,8	43,426	11	15	Cell organization and biogenesis<br>Cellular component movement<br>Metabolic process
21	XP_013972063.1	Keratin, type I cytoskeletal 10 isoform X1	62,2	32,416	13	17	Cell organization and biogenesis<br>Cellular component movement<br>Metabolic process

22	NP_001003026.1	Serum albumin precursor	68,6	21,801	4	5	Cell communication<br>Regulation of biological process<br>Response to stimulus<br>Transport
23	XP_022261847.1	Integrin beta-1 isoform X2	88,1	21,363	8	12	Cell differentiation<br>Cell growth<br>Cell organization and biogenesis<br>Cellular component movement<br>Metabolic process<br>Regulation of biological process<br>Response to stimulus<br>Transport
24	XP_005619977.3	Profilin-1	12,7	20,349	3	33	Cell organization and biogenesis<br>Regulation of biological process
25	XP_536047.2	Isocitrate dehydrogenase [NADP] cytoplasmic	46,8	17,725	5	14	Metabolic process<br>Regulation of biological process<br>Response to stimulus
26	NP_001003316.1	Ras-related protein Rab-7a	23,5	17,495	5	28	Cell organization and biogenesis<br>Cellular homeostasis<br>Metabolic process<br>Regulation of biological process<br>Response to stimulus<br>Transport

27	P02648.2	Apolipoprotein A	30,2	16,086	2	6	Cell organization and biogenesis<br>Metabolic process<br>Regulation of biological process<br>Response to stimulus<br>Transport
28	NP_001332964.1	Keratin, type II cytoskeletal 5	62,8	10,757	3	5	Metabolic process
29	XP_005621078.1	Ras-related C3 botulinum toxin substrate 1 isoform X1	23,5	10,594	4	17	Cell organization and biogenesis<br>Cellular component movement<br>Regulation of biological process<br>Response to stimulus
30	XP_536861.2	Guanine nucleotide-binding protein G(I)/G(S)/G(T) subunit beta-2	37,3	7,729	3	9	Regulation of biological process<br>Response to stimulus
31	NP_001003277.2	Ras-related protein rab-10	22,5	4,884	2	12	Cell differentiation<br>Cell organization and biogenesis<br>Regulation of biological process<br>Response to stimulus<br>Transport
32	NP_001003263.1	Guanine nucleotide-binding protein G(s) subunit alpha	45,6	4,101	2	7	Regulation of biological process<br>Response to stimulus

33	XP_534923.1	Peptidyl-prolyl cis-trans isomerase FKBP4 isoform X1	51,5	2,639	2	4	Cell organization and biogenesis<br>Metabolic process<br>Regulation of biological process<br>Response to stimulus

List of specific proteins in cLSC exosomes and canine adipose tissue MSC exosomes							
**Number**	**Accession**	**Description**	**MW [kDa]**	**Score**	**Peptides**	**Coverage [%]**	**Biological Process**
1	XP_005621076.1	Actin, cytoplasmic 1 isoform X1	44,8	236,41	23	53	Cell organization and biogenesis
2	XP_532060.4	Tubulin beta chain	49,6	98,279	18	47	Cell organization and biogenesis
3	NP_001184045.1	Elongation factor 1-alpha 1	50,1	70,419	11	27	Metabolic process
4	NP_001002961.1	Annexin A2	38,6	59,838	16	45	Regulation of biological process
5	NP_001003392.1	Keratin, type II cytoskeletal 1	63,8	43,426	11	15	Cell organization and biogenesis<br>Cellular component movement<br>Metabolic process
6	XP_013972063.1	Keratin, type I cytoskeletal 10 isoform X1	62,2	32,416	13	17	Cell organization and biogenesis<br>Cellular component movement<br>Metabolic process
7	NP_001003026.1	Serum albumin precursor	68,6	21,801	4	5	Cell communication<br>Regulation of biological process<br>Response to stimulus<br>Transport

8	XP_536047.2	Isocitrate dehydrogenase [NADP] cytoplasmic	46,8	17,725	5	14	Metabolic process<br>Regulation of biological process<br>Response to stimulus
9	NP_001003316.1	Ras-related protein Rab-7a	23,5	17,495	5	28	Cell organization and biogenesis<br>Cellular homeostasis<br>Metabolic process<br>Regulation of biological process<br>Response to stimulus<br>Transport
10	P02648.2	Apolipoprotein A	30,2	16,086	2	6	Cell organization and biogenesis<br>Metabolic process<br>Regulation of biological process<br>Response to stimulus<br>Transport
11	NP_001332964.1	Keratin, type II cytoskeletal 5	62,8	10,757	3	5	Metabolic process
12	XP_005621078.1	Ras-related C3 botulinum toxin substrate 1 isoform X1	23,5	10,594	4	17	Cell organization and biogenesis<br>Cellular component movement<br>Regulation of biological process<br>Response to stimulus
13	NP_001003277.2	Ras-related protein rab-10	22,5	4,884	2	12	Cell differentiation<br>Cell organization and biogenesis<br>Regulation of biological process<br>Response to stimulus<br>Transport

14	XP_534923.1	Peptidyl-prolyl cis-trans isomerase FKBP4 isoform X1	51,5	2,639	2	4	Cell organization and biogenesis<br>Metabolic process<br>Regulation of biological process<br>Response to stimulus
