# Supplementary material for: Characterization of the secretory profile and exosomes of limbal stem cells in the canine species
Source: PLoS One. 2020 Dec 29;15(12):e0244327. doi: 10.1371/journal.pone.0244327 (PMC7771867; doi:10.1371/journal.pone.0244327)
Supplement: S1 Raw images — (PDF) [file pone.0244327.s003.pdf]

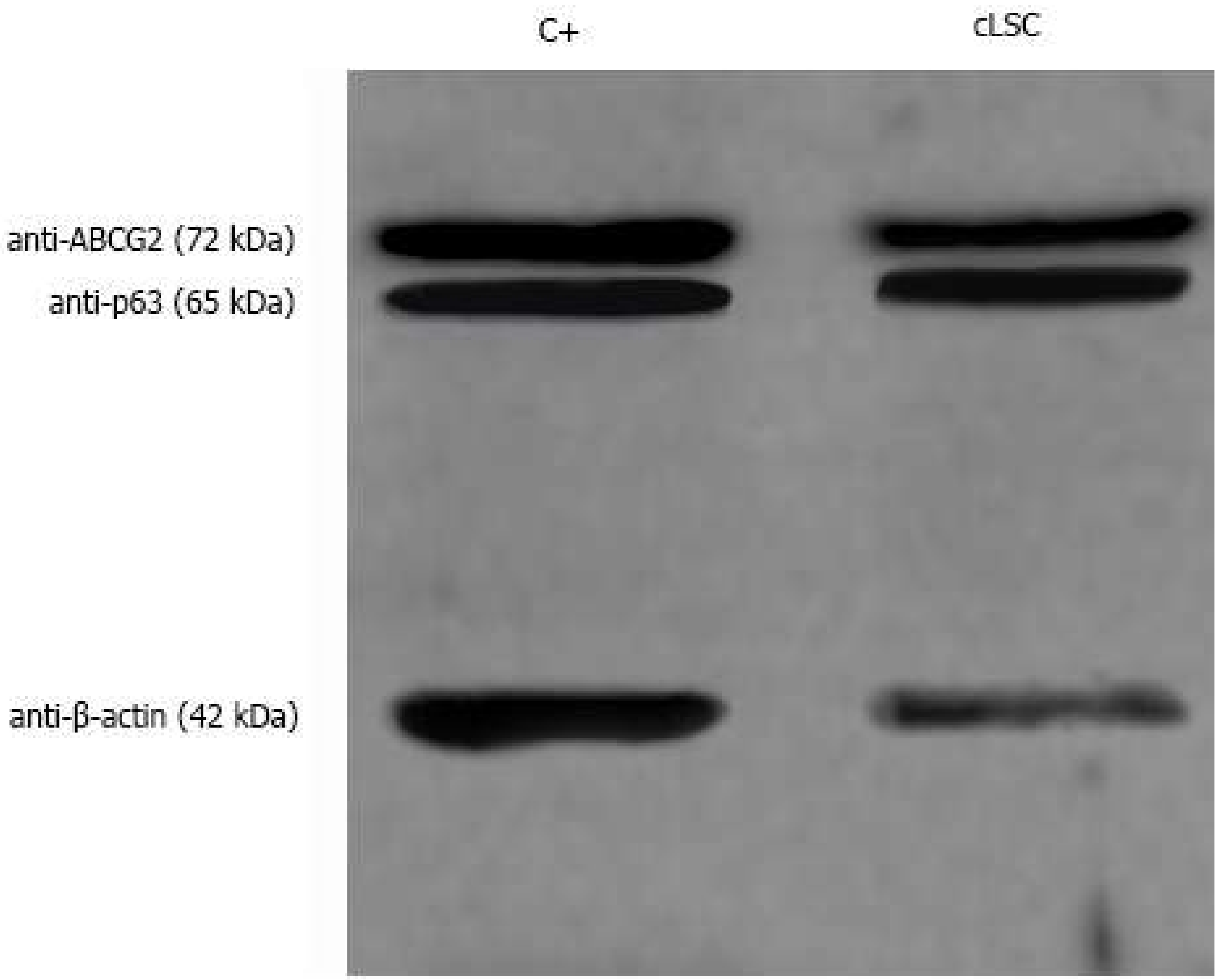

Exposure time = 20 seconds

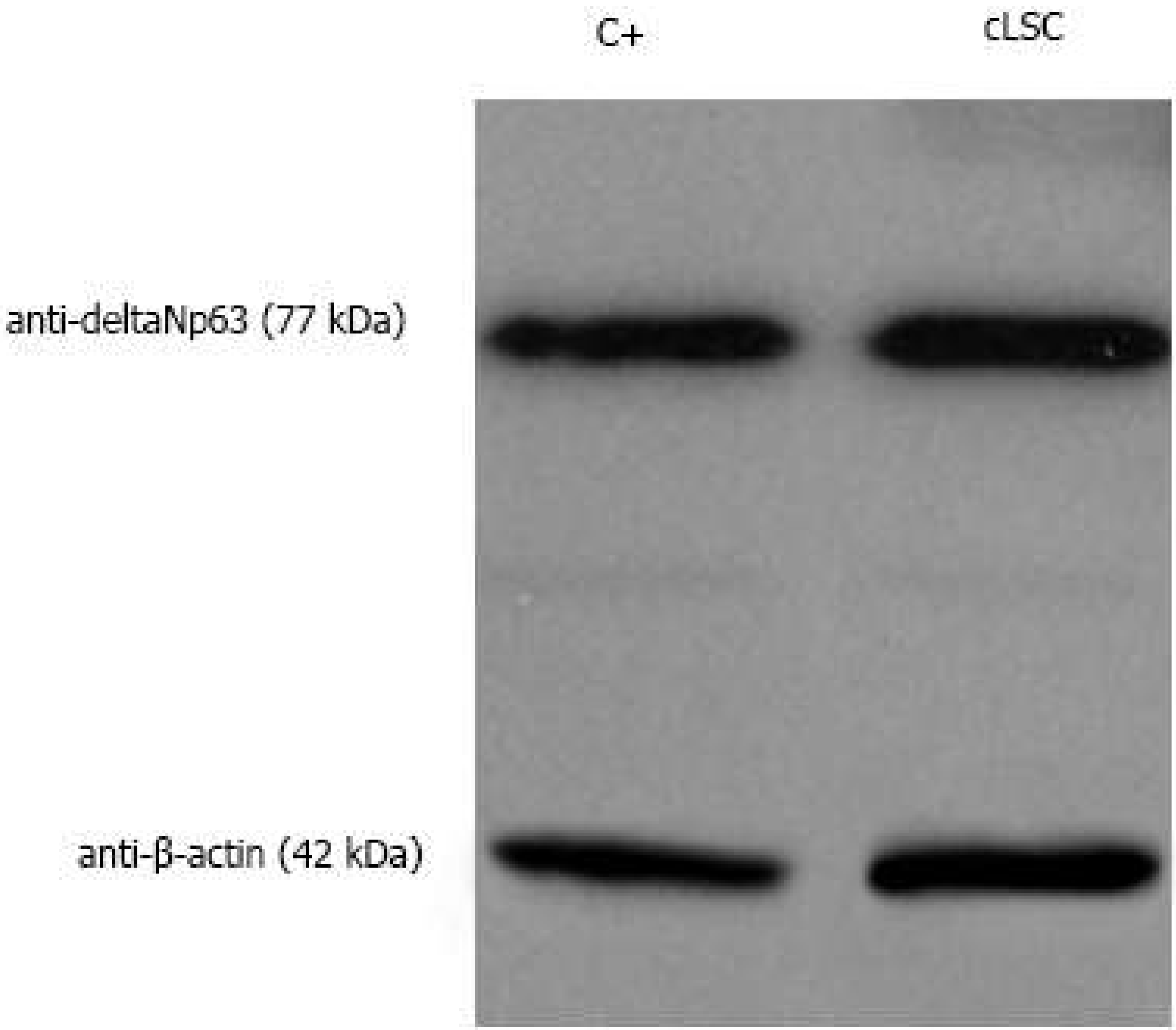

Exposure time = 20 seconds

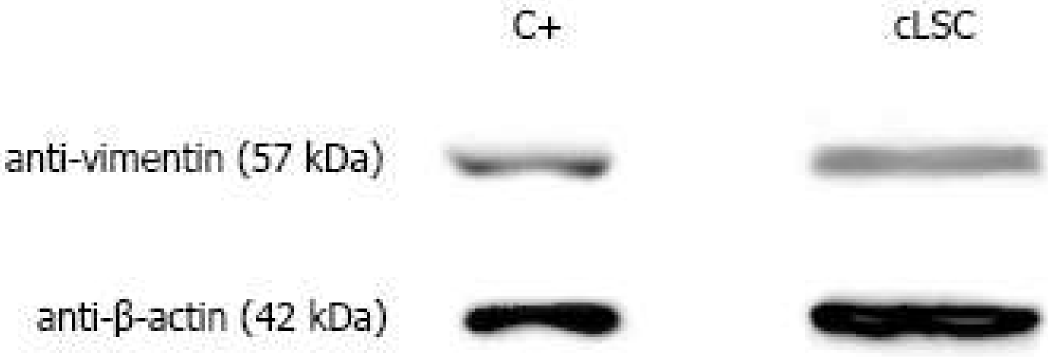

Exposure time = 20 seconds

Original WB for ALIX and TSG101

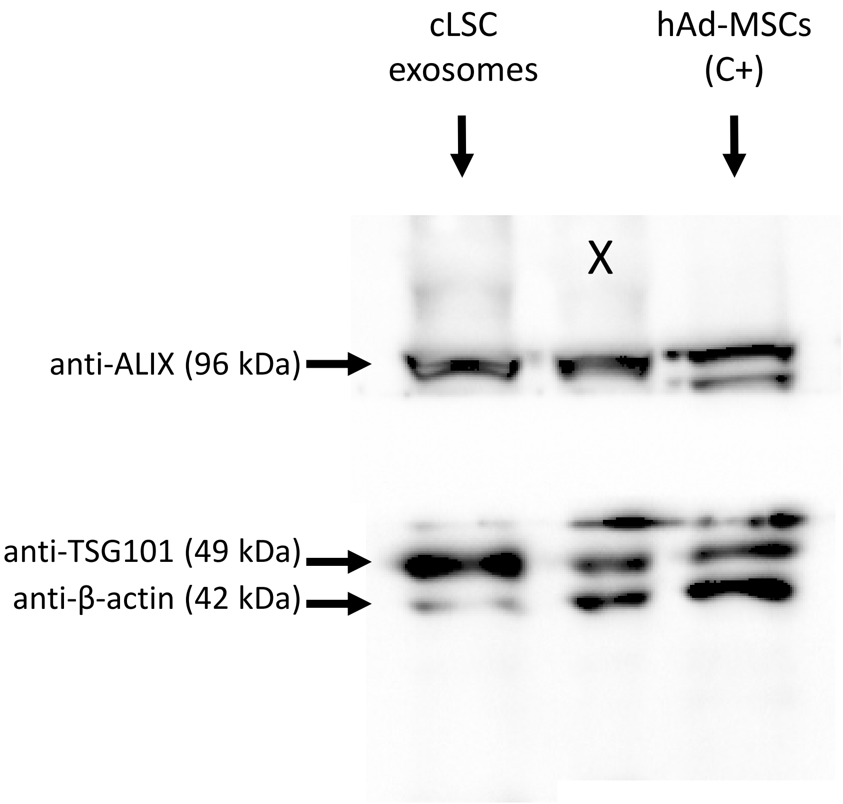

Original WB for Calnexin

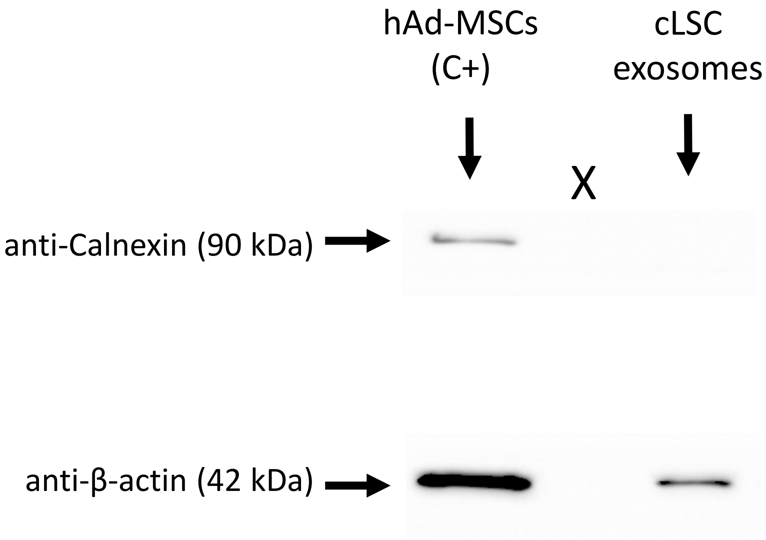

Exposure time = 20 seconds
